# A nationwide assessment of plastic pollution in the Danish realm using citizen science

**DOI:** 10.1038/s41598-020-74768-5

**Published:** 2020-10-20

**Authors:** Kristian Syberg, Annemette Palmqvist, Farhan R. Khan, Jakob Strand, Jes Vollertsen, Lauge Peter Westergaard Clausen, Louise Feld, Nanna B. Hartmann, Nikoline Oturai, Søren Møller, Torkel Gissel Nielsen, Yvonne Shashoua, Steffen Foss Hansen

**Affiliations:** 1 Department of Science and Environment, Roskilde University, Roskilde, Denmark; 2 Department of Bioscience, Aarhus University, Aarhus, Denmark; 3 Department of Civil Engineering, Aalborg University, Aalborg, Denmark; 4 Department of Environmental Engineering, Technical University of Denmark, Lyngby, Denmark; 5 Technical University of Denmark, National Institute of Aquatic Resources, Lyngby, Denmark; 6 Environmental Archaeology and Materials Science, National Museum of Denmark, Copenhagen, Denmark

**Keywords:** Environmental sciences, Environmental social sciences

## Abstract

Plastic pollution is considered one of today’s major environmental problems. Current land-based monitoring programs typically rely on beach litter data and seldom include plastic pollution further inland. We initiated a citizen science project known as the Mass Experiment inviting schools throughout The Danish Realm (Denmark, Greenland and the Faeroe Islands) to collect litter samples of and document plastic pollution in 8 different nature types. In total approximately 57,000 students (6–19 years) collected 374,082 plastic items in 94 out of 98 Danish municipalities over three weeks during fall 2019. The Mass Experiment was the first scientific survey of plastic litter to cover an entire country. Here we show how citizen science, conducted by students, can be used to fill important knowledge gaps in plastic pollution research, increase public awareness, establish large scale clean-up activities and subsequently provide information to political decision-makers aiming for a more sustainable future.

## Introduction

Plastic pollution pervades our environment^1,2^ and is considered one of the major challenges facing global society today^3,4^. The majority of environmental plastic pollution stems from ineffective waste handling, with the most important source likely being single use plastics that are either accidently lost or deliberately discarded directly into the environment^1,5^. It is estimated that 4–12 million tons of plastic are lost to the environment every year^6^. If we continue “business as usual”, as much as 12,000 million metric tons of plastic waste could be landfilled or lost to the environment by 2050^1^. According to an expert group under Science Advice for Policy by European Academics (SAPEA), risks to the environment posed by microplastics (plastic particles smaller than 5 mm) are today localized in specific hotspot areas, whereas widespread risks could be “more likely than not” within a century if pollution patterns do not change^7^ highlighting the importance of societal actions.

The increasing levels of plastic pollution have resulted in substantial societal awareness^8,9^. Citizens worldwide are taking action to mitigate plastic pollution^10–13^ at the same time as an increasing number of policy initiatives are developed to target sources and potential solutions^14^. Large scale plastic sampling campaigns such as “International Coastal Cleanup” and “World Cleanup Day” have had great success in engaging citizens all over the globe to collect litter for a cleaner environment^15^ and a Citizen Science project has recently been utilized to analyze plastic pollution in the arctic part of Norway^16^. The European Environmental Agency’s (EEA) “Marine Litter Watch” (MLW) program utilizes Citizen Science projects to inform policy-making, by organizing collection of litter items with a procedure based on the EU’s Marine Strategy Framework Directive. The procedure allows comparison between surveys completed by communities of citizens and official monitoring data. Until today the MLW program has focused solely on beach litter, neglecting data describing plastic pollution further inland^17^. This monitoring approach thus results in significant uncertainties and knowledge gaps in the amounts, composition and distribution of plastic pollution in various terrestrial nature types, such as road side ditches, forests and parks as mainly beach litter data have been used to monitor plastic pollution^18^. Filling these knowledge gaps may have great implications for risk assessment and risk management. For example transport between organisms in terrestrial food-webs relevant for human consumption^19^ may impact human exposure potential to plastic particles. As a consequence the potential impacts of plastic pollution in terrestrial ecosystems has been suggested as a focal point for future research^20^.

To address these knowledge gaps and generate public awareness, we initiated a citizen science project known as the “Mass Experiment” in close collaboration with the Danish National Center for Science Education, Astra. We asked school classes throughout The Danish Realm (Denmark, Greenland and the Faeroe Islands) to collect litter samples and document plastic litter locally during the 3-week period September 16th to October 11th 2019, making the Mass Experiment the first scientific survey of plastic litter to cover an entire country, namely Denmark.

## Results

### First full national survey of plastic pollution

**Figure 1:**
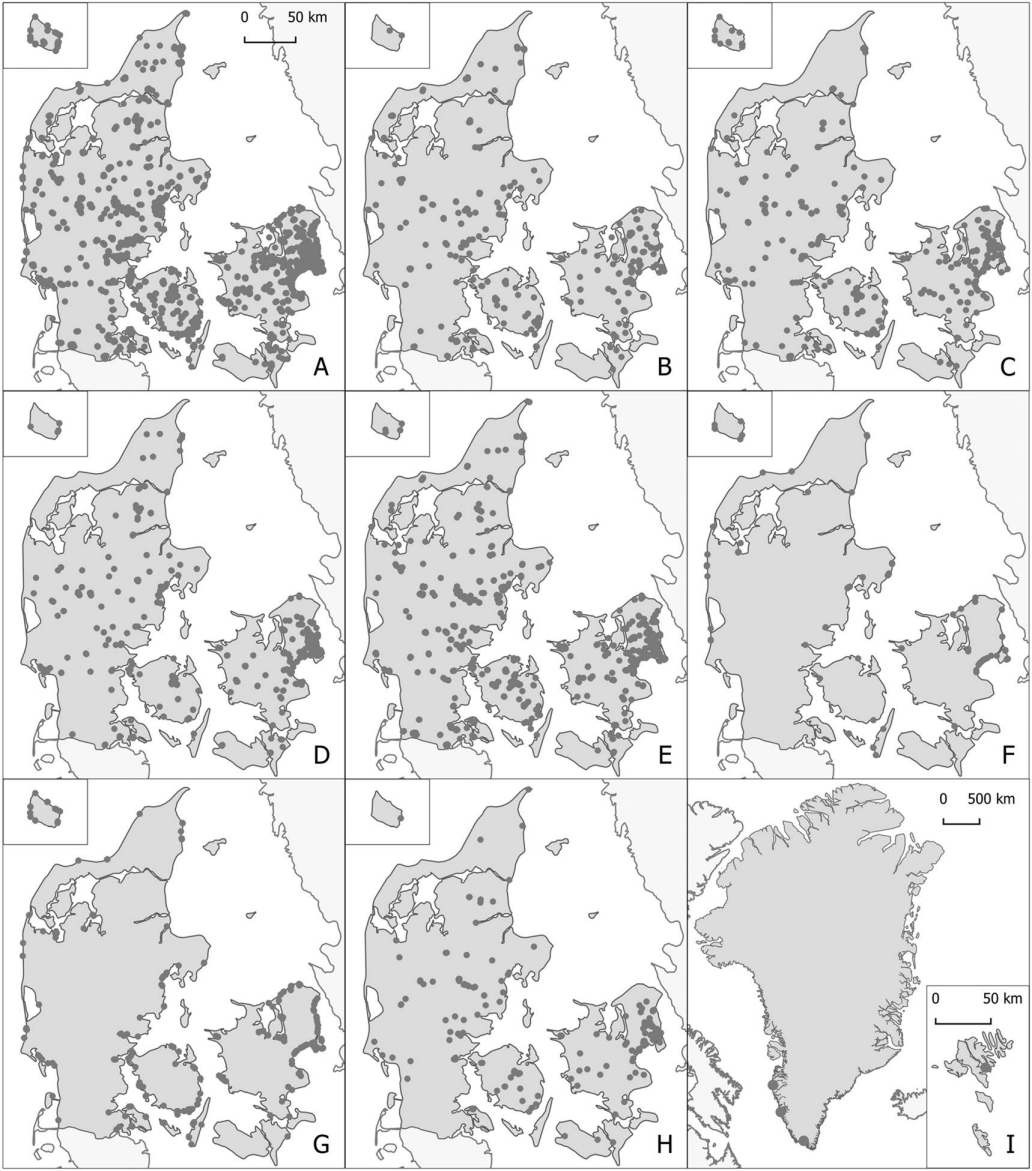
Cover of Denmark (national survey). The nine maps provide an overview of the locations for the 3,452 surveys conducted in the Mass Experiment. One spot may represent the GPS-coordinate from several survey carried out in close proximity. The legends on the map are as follow. (**A**) All surveys; (**B**) Rural roadside (**C**) Forest path (**D**) Park; (**E**) ditch along roadside; (**F**) Dune; (**G**) Beach; (**H**) Stream and lake side and (**I**) Arctic/Subarctic (i.e., Greenland and Faroe islands).

Fifty-seven thousand 6–19 year old students, representing classes from private and public elementary schools as well as high schools, participated in the project. This corresponds to approximately 1% of the total population of The Danish Realm. In total, 374,082 plastic litter items were collected on 3542 transects (i.e. a 100-m zone with varying width), distributed over eight different nature types (Fig. 1). Sampling was conducted over three weeks in the fall. Weather conditions were comparable over the entire sampling period, with daily rainfalls varying between 0–0.4 mm (mean ± SD: 0.127 mm ± 0,128 mm) during sampling hours (9.00–18.00). Comparing activity on the day with highest rainfall (0.04 mm) with the four days with no rainfall can indicate whether weather conditions influenced the sampling. On the rainiest day, 313 samples were collected containing, on average, 119.3 items of plastic. On the four days with no rain 108, 163, 556 and 110 samples were collected with an average number of plastic items of 103.9, 101.7, 92.2 and 110.5, respectively. This indicates that weather had little impact on the sampling activities.

Samples were sorted into 22 different categories of plastic, yielding 77,990 unique data points (Table 1).

**Table 1:** Overview of data.

Category	Plastic items	% of total	Category	Plastic items	% of total
1. Shopping bags	9264	2.5	12. Straws and stirrers	6304	1.7
2. Small plastic bags	28,011	7.5	13. String and cord	6740	1.8
3. Drink bottles (≤ 0.5 l)	4550	1.2	14. Nets and pieces of net < 50 cm	1913	0.5
4. Drink bottles (> 0.5 l)	1260	0.3	15. Nets and pieces of net > 50 cm	694	0.2
5. Food containers incl. fast food Containers	6428	1.7	16. Plastic pieces 2.5 cm > < 50 cm	67,387	18.0
6. Plastic caps/lids; drinks	8545	2.3	17. Polystyrene pieces 2.5 cm > < 50 cm	17,316	4.6
7. Plastic caps/lids; unidentified	6338	1.7	18. Cotton bud sticks	1264	0.3
8. Cigarette butts and filters	112,018	29.9	19. Sanitary towels/panty liners/backing Strips	2745	0.7
9. Crisp packets/sweet wrappers	48,299	12.9	20. Other plastic/polystyrene items (identifiable)	24,800	6.6
10. Cups and cup lids	7539	2.0	21. Balloons and balloon strings and sticks	2397	0.6
11. Cutlery and trays	3631	1.0	22. Other rubber pieces	6669	1.8

Overview of the plastic items collected in the Mass Experiment. Total amount of items collected under each of the 22 categories are shown as well as the percentage contribution of the total of each plastic item. The plastic categories are numbered 1–22 and the G-numbers in brackets refer to the labeling in the JRC litter guide that formed the basis for selection of the 22 categories.

All nature types were well covered in the Mass Experiment spanning from 169 to 931 transects, the exception being the arctic/subarctic nature type (Greenland and Faeroe Islands) where only 14 transects were sampled (Table 2).

**Table 2:** Distribution and plastic litter content of surveys (transects). Overview of the Mass Experiment.

Nature types	Number of surveys	% of total surveys	Number of plastic items (n)	% of total plastic items	Lowest number (n)	Highest number (n)
Beach	430	12.1	39,150	10.5	0	1401
Dune	169	4.8	23,210	6.2	0	3253
Forest path	596	16.8	34,868	9.3	0	665
Stream and lake sides	257	7.3	22,210	5.9	0	861
Ditch along roadside	931	26.3	113,553	30.4	0	1606
Rural roadside	450	12.7	28,452	7.6	0	627
Park	695	19.6	110,503	29.5	0	3322
Arctic/Subarctic	14	0.4	2,136	0.6	41	427
Total	3542	100	374,082	100		

For each of the eight nature types number of surveys and surveys as percent of total is presented. Number of plastic items and their percent share of the total is further presented as well as lowest and highest number of plastic items found in a single survey.

On average, 105.4 ± 3.1 pieces of plastic (mean ± 95% confidence interval (CI)) were collected for every transect. Fewer than 2% of the transects (n = 66) did not contain any plastic, illustrating that plastic pollution is present almost everywhere in Denmark. With data from more than 95% of Danish municipalities, the dataset provides a broad national coverage both geographically and in terms of nature types (Fig. 1). Coverage of the Faroe Islands and Greenland was sparser, generating data for single locations rather than the full landmasses (Fig. 1I).

### Distribution of plastics in the seven nature types

All of the 22 different types of plastic items were represented in each of the seven different nature types in Denmark (supplementary information 1).

Based on total quantities of plastic items, almost one third (30.4%) were collected in ditches along roadsides, whereas the lowest amount were found along streams and lakes (5.9%) (Table 2). The largest variation in pollution levels within a single nature type were found in parks where seven samples contained no plastic and sixteen contained more than 1000 items. The largest number of pieces collected in a single survey was 3322 plastic items, also found in a park. All data are available at: https://doi.org/10.5281/zenodo.3886974.

**Figure 2:**
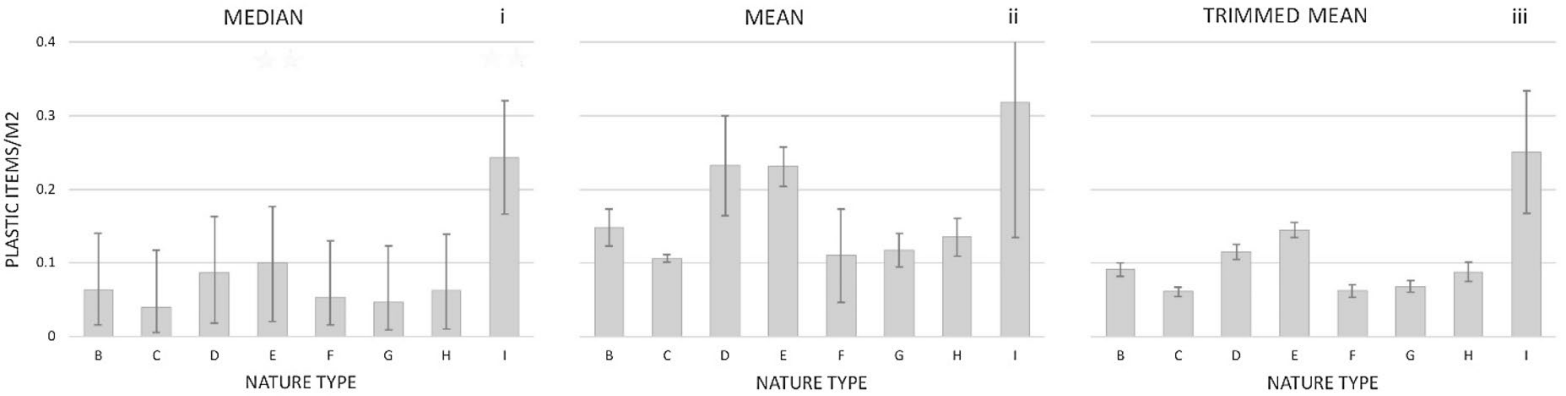
Prevalence in nature types. Concentrations of plastic items in each of the eight nature types presented as (**i**): median ± MAD; (**ii**): mean ± SD and (**iii**): trimmed mean (10%) ± SD. Concentrations are provided as plastic items/m^2^ and were assessed on the basic of all 22 categories pooled. Nature type numbers correspond to (**B**): Rural roadside, (**C**): forest path, (**D**): park, (**E**): ditch along roadside, (**F**): dune, (**G**): beach, (**H**): stream and lake side and (**I**): arctic/subarctic.

Since the number of samples differed between nature types and the sampled area differed between surveys, abundance per area provides better foundation for comparisons. When comparing numbers based on measure of central tendency of plastic for the seven nature types in Denmark, we found that ditches had the highest median values (0.10 ± 0.080 items m^−2^), whereas the lowest was found in forests (0.040 ± 0.035 items m^−2^). Beaches and dunes had the second and third lowest median values (0.047 ± 0.039 items m^−2^ and 0.053 ± 0.038 items m^−2^ for beaches and dunes respectively), indicating that plastic pollution collected at beaches might not represent a worst-case scenario for other terrestrial compartments (Fig. 2i).

Concentrations of plastic items found in ditches were significantly higher than for all other nature types (except the Arctic/subarctic) (*p* < 0.05) indicating that this nature type is the overall most polluted in The Danish Realm. The results are slightly different if measures of central tendency are estimated by mean values (Fig. 2ii). Ditches (0.231 ± 0.42 items m^−2^) is then just surpassed by parks (0.232 ± 0.905 items m^−2^), even though the two values are not significantly different (*p* > 0.05). The difference in central tendencies expressed as median and mean reflects that the datasets are not normally distributed (see supplementary information 2 for analyses for normality). The few park samples with very high number of plastic items affect the calculation of the mean for this nature type, because means are more sensitive to extreme values than median values. This is further highlighted in the trimmed mean 10% estimation, where the dataset is corrected for the most extreme values, since the pattern is approaching that of the median to a higher degree (e.g. 0.145 ± 0.162 items m^−2^ and 0.115 ± 0.144 items m^−2^ for Ditches and Parks, respectively (Fig. 2iii)). The Arctic/subarctic had the highest median (0.228 ± 0.078 items m^−2^) and mean (0.318 ± 0.351 items m^−2^) values of all nature types, and the Arctic/subarctic was furthermore the only nature type where plastic was found in all samples. The total numbers of plastic items ranged between 41 and 427 (Table 2). This could indicate that the Arctic/subarctic regions of The Danish Realm are the most polluted. However, it is important to note that these observations are based on a low number of samples (n = 14) (Table 2) and that the Greenlandic population is very unevenly distributed geographically (i.e., only a small part of Greenland is populated).

### Distribution of plastic types

**Figure 3:**
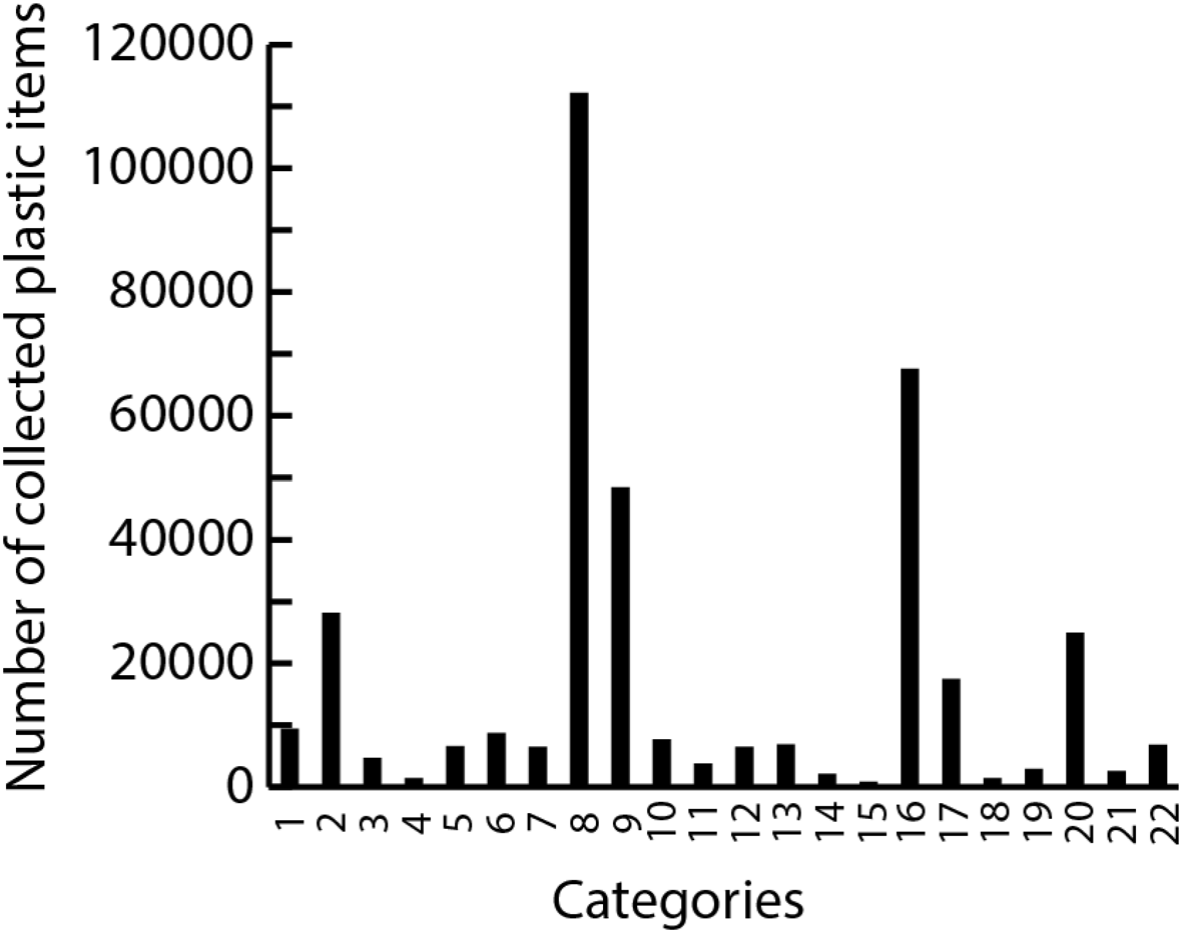
Distribution of plastic item among the 22 categories. Number of plastic items distributed along the 22 plastic categories for the total dataset (see supplementary information 1 for distribution in each nature type). The numbers correspond to (**1**) Shopping bags, (**2**) Small plastic bags, (**3**) Drink bottles (≤ 0.5 l), (**4**) Drink bottles (> 0.5 l), (**5**) Food containers incl. fast food containers, (**6**) Plastic caps/lids; drinks, (**7**) Plastic caps/lids; unidentified, (**8**) Cigarette butts and filters, (**9**) Crisp packets/sweet wrappers, (**10**) Cups and cup lids, (**11**) Cutlery and trays, (**12**) Straws and stirrers, (**13**) String and cord, (**14**) Nets and pieces of net < 50 cm, (**15**) Nets and pieces of net > 50 cm, (**16**) Plastic pieces 2.5 cm >  < 50 cm, (**17**) Polystyrene pieces 2.5 cm >  < 50 cm, (**18**) Cotton bud sticks, 19. Sanitary towels/panty liners/backing strips, 20. Other plastic/polystyrene items (identifiable), 21. Balloons and balloon strings and sticks, 22. Other rubber pieces.

The most commonly found plastic litter items were cigarette butts followed by plastic pieces and candy/chips wrappings (Table 1, Fig. 3).

These three categories account for 29.9%, 18% and 12.9% of the plastic items collected, respectively, and thus together constituted more than 60% of all plastics sampled. Nets larger than 50 cm was the least commonly found plastic item (n = 694). The five least represented categories of plastics items, namely large nets, cotton bud sticks, small bottles, small nets and balloons/-strings and sticks constituted altogether only 2% of the total number collected.

In general, the dominant types of plastic items were the same across many of the nature types, although the numbers of each varied with location (See supplementary information 1). For ditches, parks, streams, and lakes, the 6 most commonly collected items were, from highest to lowest, cigarette butts, plastic pieces < 50 cm, crisp/candy wrappers, small plastic bags, other identifiable plastic/polystyrene items, foamed and expanded polystyrene pieces < 50 cm. Along rural roadsides, a similar pattern was observed, but small plastic bags and other identifiable plastic/polystyrene items had swapped places. On forest paths, cigarette butts took third place while carrier bags were sixth. Around dunes, foamed polystyrene pieces < 50 cm were found to such an extent that they are surpassed only by cigarette butts and plastic pieces < 50 cm. The same was observed for strings and cord as well as foamed polystyrene pieces < 50 cm on beaches. In the Arctic/subarctic nature type, strings and cord were the eight most abundant litter types (n = 128), accounting for 6% of all plastic items, probably reflecting the importance of fishing activities in these regions.

### Public awareness

Besides collecting information about amounts and types of plastic pollution from the most common types of plastic litter found in different nature types, the Mass Experiment contributed significantly to awareness raising and education of the 57,000 students active in the project. The focused teaching materials developed and distributed to the responsible teachers of each school class two months prior to the beginning of the sampling period, provided them with ample time to prepare and evaluate activities and to clarify any potential questions in advance. The teaching material included the following YouTube videos: 1. An introduction to the Mass Experiment, 2. An introduction to the most common polymer types for age group 6–13 years, 3. An introduction to the most common polymer types for age group 13–19 years and 4. An Introduction to the sampling and categorizing of plastic items. In addition, teachers’ guides, a sampling protocol, a cloth with pictures of the 22 item categories to facilitate on-site sorting and categorization of the samples, and a poster explaining the major sources of plastic pollution in Denmark were supplied. The Mass Experiment also included a protocol for an additional, optional activity, designed to identify the polymer components of the plastic items using simple techniques such as density separation and burning tests. This additional protocol was designed mainly as an educational supplement for the older participants. Five additional videos and a protocol for this part was distributed as well. (All materials in Danish may be accessed without payment at https://naturvidenskabsfestival.dk/tildinundervisning/masseeksperiment-2019-plastforurening-i-vand).

The Mass Experiment attracted significant public attention and initiated societal debate about sources and impact of plastic pollution with 140 registered media mentions in connection with the collecting period in September 2019 and again immediately following the release of the results (January 27–30, 2020). The media coverage comprised 11 TV stories, 41 newspaper articles, 24 radio interviews and discussions, 59 web-based media events and 5 news distributing media (e.g. Reuters). The media distributors were international organizations such as Reuters, Danish newspapers, as well as the two major Danish TV stations. The news coverage generated 1,334 registered social media engagements measured as likes, mentions, retweets, and comments, on Twitter, Instagram and Facebook. A word cloud was made to identify the most important words associated with the media coverage. Among the highlighted words were “scientific methods”, “citizen science”, “great teaching”, “Danish plastic pollution” and “Danish nature and plastic pollution”, illustrating that the majority focused on both the scientific and the societal aims of the Mass Experiment and the learning outcomes. (Supplemental Material 3 for full overview of the coverage).

## Discussion

Out of the 3542 transects studied in the Mass Experiment, less than 2% contained no plastic items, illustrating that plastic pollution was found throughout all the investigated nature types. This highlights the need to increase focus on other compartments than the marine environment/beaches in future environmental monitoring programs. Concentrations ranged from 0.04 to 0.1 plastic items m^−2^ which is in the same range as riverine concentrations reported in Germany, where the interquartile range was 0–0.57^21^. Ditches were the most polluted nature type in the Mass Experiment. Previous comparable surveys have indicated that littering by pedestrians and motorists might be the major source of plastic pollution^22^, which could explain why the highest concentration were found where transport activities take place. Many of the schools reporting high values, investigated as potential outliers, explained that their sampling locations were places where people tends to gather, stay or wait, e.g. some parks or bus stops, indicating that the highest single concentrations were associated with areas of social activities. Since plastic pollution is not static but typically move moves through the environment, and ultimately end up in the marine compartment, our data illustrate that future monitoring efforts should include nature types with high human activities, in order to access environmental plastic pollution as a whole, such as ditches along roadsides and parks.

There are some exceptions to the general trend that we observed regarding the most common plastic items found in the different nature types, which amongst others, includes a more frequent distribution of strings and cord on beaches. One possible reason for the higher amounts of strings and cords on beaches could be that they were primarily lost to nature in connection with sailing and fishing activities. In the dunes, pieces of foamed polystyrene (< 50 cm) are only surpassed by cigarette butts and plastic pieces (< 50 cm). The large amounts of pieces of foamed polystyrene may originate from e.g. fragmented insulating, packaging materials and floats related to fishing. Forest paths contained measurably fewer cigarette butts and was only found third most in contrast to being the most frequently collected plastic item in all other nature types. There may be several reasons for this. In Denmark, smoking is prohibited in coniferous forests, grassy forest floor, heather areas or young stands between March 1 and October 31, which account for at least 48% of the Danish forests. Furthermore, public and private owners of forests are empowered to prohibit smoking in accordance with the Nature Conservation Act and the Access Order^23^. Overall, forest paths were the least polluted nature type studied in the Mass Experiment. The reason might be, that social values and norms are stronger drivers than sociodemographic distributions^24^, and that forests are more highly valued as an important nature type and therefore more worthy of protection from littering by users than, for example, ditches along roadsides.

The data from the Mass Experiment provided a unique opportunity to compare data from a full national survey with marine litter data collected on European beaches within the past decade. Cigarette butts were the most common plastic item found in the Mass Experiment corresponding to an average of 31.6 ± 2.8 butts per sample. This finding fits those of other monitoring surveys including those from Danish beaches^15,17,22,25^, and could illustrate that discarding cigarette butts may not be considered littering by many smokers^26^, because surveys of beaches that attract few visitors find fewer cigarette butts. Surveys on Danish reference beaches with limited amount of visitors did not find cigarette butts to be among the most common plastic items^27^. This indicates that direct littering is the main source of pollution with these plastic items, and that targeted efforts are needed to change the littering behavior of smokers.

The finding that crisp/sweet wrappers were the third largest litter type in the Mass Experiment also fits patterns observed throughout Europe^28^. Together with plastic pieces and small plastic bags, cigarette butts and sweet/crisp wrappers constituted almost 70% of all plastic items collected. Both plastic pieces and cigarette butts were among the smallest types of litter collected in the Mass Experiment. These are typically harder to find that larger types of litter. Their high abundance together with the comparability with other findings^21^ confirms that the participants were able to follow the developed protocol and conduct thorough collections of litter within the transects.

In May 2019, the Single-Use Plastics Directive (SUP Directive) was formally adopted in the EU to reduce the impact of certain plastic products on the environment and to prevent further accumulation of marine litter^29^. Measures among other things includes, phasing out specific single-use plastic items such as cutlery, plates, straws, cups and food packaging for take-away meals and cotton bud sticks^29^. Single use plastic items dominated our samples, which confirmed the general scientific findings supporting the SUP directive, with some exceptions in pollution patterns (Table 3).

**Table 3:** Plastic categories and their relation to the Single use plastic (SUP) directive.

Categories	Corresponding G number	Rank in Mass experiment	SUP top ten (corresponding G number)
Shopping bags	G3	7	No. 6 Plastic bags (G3, G4)
Small plastic bags	G4	4	See G3
Drink bottles (≤ 0.5 l)	G7	15	No. 1 Drinks bottles, caps and lids (G7,G8,G21)
Drink bottles (> 0.5 l)	G8	21	See G7
Food containers incl. fast food Containers	G10	12	No. 10 Food containers including fast food packaging (G10)
Plastic caps/lids; drinks	G21	8	See G7
Plastic caps/lids; unidentified	G23	13	See G7
Cigarette butts and filters	G27	1	No. 2 Cigarette butts (G27)
Crisp packets/sweet wrappers	G30	3	No. 4 Crisp packets/sweet wrappers
Cups and cup lids	G33	9	No. 8 Drinks cups and cup lids (G33)
Cutlery and trays	G34	16	No. 7 Cutlery, straws and stirrers (G34, G35)
Straws and stirrers	G35	14	See G34
String and cord	G50	10	Fishing gear*
Nets and pieces of net < 50 cm	G53	19	Fishing gear*
Nets and pieces of net > 50 cm	G54	22	Fishing gear*
Plastic pieces 2.5 cm > < 50 cm	G79	2	N.A.**
Polystyrene pieces 2.5 cm > < 50 cm	G82	6	N.A **
Cotton bud sticks	G95	20	No 3. Cotton bud sticks (G95)
Sanitary towels/panty liners/backing Strips	G96	17	No 5. Sanitary applications
Other plastic/polystyrene items (identifiable)	G124	5	N.A **
Balloons and balloon strings and sticks	G125	18	No 9. Balloons and balloon sticks (G125)
Other rubber pieces	G134	11	N.A. **

Each of the 22 categories used in the Mass Experiment. The corresponding G number refers to the numbering of the category in the European litter watch program. Rank in the Mass Experiment based on collected numbers as well as their corresponding ranking in the surveys laying the foundation for the single use plastic directive are provided for comparison.

*Fishing gear is targeted under the SUP Directive but not as one of the top ten SUP items.

**N.A.: not applicable. Refers to items that are not directly relevant for the SUP directive.

In the Mass Experiment, plastic bottles were ranked 15 and 21 out of 22 categories for small (≤ 0.5 L) and large bottles (> 0.5 L), respectively. In contrast these categories typically ranks in the top-ten most found items on European beaches^28,30^. This difference may be attributed to the effective bottle deposit/refund system first established in Denmark in 1922 as a response to resources deficiency after world war one, and expanded to cover plastic bottles in the early 1990s^31^, as it ensures that used bottles gain monetary value. Our findings are in accordance with data collected in a different sampling campaign, namely the Danish Society for Nature Conservation (DSNC) annual litter waste clean-up and collection week. Out of the 110,000 beverage cans that were collected from 25.-31. April of 2019, the majority were imported and therefore outside the Danish deposit-return system^25^. Collectively, the data from the Mass Experiment together with the DSNC datadata indicates that assigning a monetary value to plastic waste items can reduce littering of selected SUPs effectively. This illustrates that the initiatives under the SUP directive can have a positive impact on plastic littering in Denmark and the rest of EU.

Plastic bags were collected in the Mass Experiment (category 1 and 2. See supplemental information 4 for pictograms of the two types of plastic bags). Lightweight plastic bags with wall thickness below 50 µm are targeted by the EU Directive on reducing the consumption of lightweight plastic carrier bags (Directive (EU) 2015/720) adopted in 2015, as an amendment to Directive 94/62/EC^32^. However, this does not include commonly use Danish plastic shopping bags (category 1 in the Mass Experiment), which are typically thicker than 50 µm. In Denmark, there is a levy on plastic shopping bags and they comprised just 2.5% of the plastic items found in the Mass Experiment. Small, thinner, plastic bags (category 2 in the Mass Experiment) do not attract the same levy in Denmark and were 3 times more abundant than the shopping bags in the Mass Experiment (Table 1). An Irish levy on plastic bags was introduced in 2002 when they comprised 5% of the litter found. By 2015 this fraction had been reduced to 0.13%^33^. The findings from the Mass Experiment in Denmark combined with experience from Ireland further confirm that charging a levy on plastic items increases their post-use value and reduces their occurrence in the environment.

Our findings illustrate that actions are needed to reduce and mitigate plastic pollution. Also, a transition in plastic consumption towards a more sustainable use in accordance with the UN sustainable development goals (SDGs) is mandatory^34^. Fritz et al.^35^ discussed how data from citizen science projects can be utilized as a non-traditional data source for the SDGs in regard to five dimensions, namely spatial, temporal, thematic, process, and data management. The Mass Experiment provides an illustrative case concerning these dimensions. The nationwide dataset provides, for the first time, a unique insight into spatial distribution of plastic pollution across all nature types in Denmark. The > 77,000 individual data points can contribute to focusing policy actions towards those plastic items that pollute the environment thereby contributing toward meeting SDG 14 and SDG 15 (life below water and land, respectively)^34^. The dataset does not yet provide information about temporal distribution of plastic pollution; however, the Mass Experiment is intended to be repeated within 5 years to assess the impact of new policy measures on some prioritized types of plastic litter items. The weight of plastic items might also be included at this point. The Mass Experiment contributes data on plastic pollution in terrestrial nature types not commonly reported, hereby addressing the thematic dimension. Our data can be characterized as tier II data informing the SDGs—i.e. data for which the sampling methodology is available but not systematically collected by countries^35^.

The Mass Experiment generated extensive public interest with more than 140 References in media and more than 1300 social media engagements, also drawing the attention of the political system in Denmark, including discussions about littering, sorting of waste and consumption of single use plastics. Information campaigns are among the measures recommended for all categories of SUP in the single use plastic impact assessment^30^. The Mass Experiment provides an example of how citizen science can facilitate a necessary debate about responsible consumption and production (SDG 12), and also contribute data and trends to motivate activity within environmental management of plastic pollution^36^. Finally, the Mass experiment contributed to educating younger generations on the importance of the transition towards a more sustainable and circular use of plastic. Bridging science and education on this scale complies well with SDG 4 on educational quality^34^. It has been shown that educating children can be an effective way to enhance the families’ and peers’ environmental awareness^37^. Although the Mass Experiment was not designed to quantify the increased awareness in the immediate networks of the participants, the significant attention reflected by the number and variety of events in published, broadcast and social media indicate its high impact on public awareness. This impact was enhanced by the fact that the participants involved directly covered approximately 1% of the Danish population.

## Methods

### Study site

The Mass Experiment was conducted throughout The Danish Realm in fall 2019. The Danish Realm consists of Denmark, the Faeroe Islands and Greenland. Denmark consists of the Jutland Peninsula and 391 islands^38^, has an area of 43,094 km^2^, and a population of almost 5.8 million inhabitants. Approximately 923,000 inhabitants were between 6 and 19 years during the Mass Experiment^39^. The Faroe Islands consist of 18 islands with a total area of 1399 km^2^ and a population of nearly 52,000^40^, whereas Greenland has an area of 2,166,086 k m^2^ and nearly 56,000 inhabitants^41^. All the countries in the Danish Realms have 9 years of mandatory compulsory education for everyone aged 6–15 years^38^. The population density is 131 person per k m^2^ in Denmark^38^, 34.5 person per k m^2^ on the Faroe Islands and 0.3 person per k m^2^ in Greenland (with an very uneven distribution). Denmark has a coast line of more than 7300 km to the North sea, The Baltic sea and the inner Danish waters (the Belt Sea, Kattegat and Oresund)^38^. The 18 islands of the Faroe Islands and Greenland have a total coastline of 1100 km^40^ and 44,087 km, respectively to the Atlantic ocean and the Arctic ocean^41^.

### Development of the sampling protocol

The scientific quality of citizen science projects is highly dependent on the development of a coherent and thorough sampling protocol and the education of the citizens involved^42^. Sampling error often occurs because participants differ in their ability to properly characterize the samples^43^. To ensure that participants had the best possibly foundation for their involvement, an educational program was developed in parallel with the sampling protocol. The sampling protocol and the educational program were developed over six months in spring 2019 in an iterative process prior to the initiation of the Mass Experiment, using teaches as peers. This procedure built upon more than ten years of experience within Astra, working with this specific participant target group in citizen science projects, as well as scientific recommendations for collection of reliable data in citizen science projects^42,43^. The protocol was made in two versions. One; one for the students and one for the teachers. The version made for the students focused on the actual sampling procedure and further explained how data should be reported. The teacher’s version was aimed at addressing potential challenges during the sampling, such as staying with the transect and collecting all litter not just the larger pieces, as well as an overview of materials needed to conduct the sampling. It further provides information about plastic pollution and the considerations behind the Mass Experiment, useful in the teaching related to participating in the Mass Experiment.

The sampling protocol was based on the EUs Joint Litter Category List^44^ modified specifically for the purpose of the Mass Experiment (Table 3). The adoption was done during the iterative process described above in order to ensure that: 1. All participants should be able to follow the protocol and categorize the collected plastic items, and 2. Ensure that the sampling could cover the entire country. The protocol was limited to 22 litter type categories, focusing solely on plastic pollution, and hence other litter material types were excluded. This modification ensured that even the youngest children aged six were able to categorize all collected items. The 22 categories were chosen based on expert judgement and aimed at covering the most important and widespread litter categories, including the top ten items defined in the single use plastic (SUP) directive^29^ (Table 3). Furthermore, the protocol was expanded to include seven different terrestrial nature types instead of focusing on beaches alone, to cover the entire country (Table 2). The Arctic/subarctic region of Greenland and the Faroe Islands were further classified as an individual eight nature type, to distinguish this specific part of the Danish Realm. The seven mainland nature types were selected through expert elicitation based on the iterative collaboration with the teachers and pupils that served as peers, as well as an evaluation of the geography of mainland Denmark.

Items were reported in numbers rather than weight. No single measurement is considered perfect, but number is regarded the best indicator for impact assessments by EU^30^. Based on the feedback from peers during the protocol development, a single way of reporting items was deemed best to avoid unnecessary uncertainties. Apart from numbers of plastic items in each of the 22 litter type categories, the following information was recorded: location (GPS coordinate), nature type, participant ID (on class level), time and date. Finally, participants were encouraged to add any additional information that they found important. To ensure standardized sampling, 3500 “sampling kits” were developed and distributed to all participating groups. The kit contained: bags to collect plastic items, latex gloves for the protection of the participants, tweezers to collect smaller items, a 2 × 1 m cloth with pictograms of all 22 item categories to sort items on-site (Supplementary information 4), protocol for sampling in two version (one for the participating student and one for the teachers), poster with sources of plastic pollution and most common polymers. Apart from the kit, all participants were provided with links to educational material, regarding both specifics to the actual sampling and plastic pollution in general. All material is available online at Astra’s homepage^45^.

### Participating classes

All primary and secondary school classes in Denmark were invited to participate. Astra has organized a mass experiment each year since 2009 with different topics, as part of the Danish science festival, and have built a comprehensive network to reach schools throughout Denmark. Advertisement started primo 2019 with information about the Mass Experiment on plastic pollution and online registration were open from 1. April. Initially the Mass Experiment was limited to the first 2,500 classes, equaling the number of sampling kits available. This was increased to 3,500 due to intensive interest in participation and made possible by a private fund donation. The 3,500 classes from primary and secondary schools comprised approximately 57,000 participants aged 6–19 years, which is about 6.2% of the Danish population in this age group and 1% of the entire Danish population^39^. The participants represented 94 out of 98 Danish municipalities, as well as locations in Greenland and the Faroe Islands. Each class had a teacher as contact person, who received the educational material and the sampling kit two months prior to the sampling weeks, allowing for dialog about questions and uncertainties related to the mass experiment. The responsible teachers included the Mass Experiment in their teaching and thus ensured that the participating children understood the protocol before sampling and reporting of data.

### The sampling

Sampling surveys took place from 16 September to 4 October in 2019. The short timespan was chosen to minimize temporal variability in the dataset^43^. Each participating class was free to decide when the specific sampling took place within the three weeks. The responsible teacher coordinated samplings, to ensure that the protocol was used throughout all phases. In the sampling protocol as well as in the explanatory youtube videos the importance of precision in collection rather than gathering all plastic litter found was emphasized. All participating teams were allowed to perform multiple surveys if these were reported independently. Surveys were performed over a 100 m transects with varying width, in order to allow for targeted sampling in the different nature types (e.g. a roadside ditch has a very different width compared to a beach). Two–three meters were suggested as potential width and the importance of reporting the total area was explicitly stressed. A link to mathematical teaching materials regarding calculation of area was further provided, so teachers of the younger students could implement this in their teaching activities related to the Mass Experiment. Samples were categorized on-site using the cloth provided in the sampling kit (Supplementary information 4). All items were subsequently collected and disposed of as litter. All data were reported directly in an online database and stored for further analyses (Supplementary information 4).

### Quality of data

There is an inherent potential risk of error and bias when many different participants collect data in a citizen science project^43^. However, the “law of large numbers” prescribe that non-systematic errors are inverse proportional with increase in data^46^, and since the Mass Experiment included approximately 77,000 individual data points, such errors were deemed to be low. One citizen science specific bias is the potentially high variability among participants due to demographics, ability, effort, and commitment^43^. It is therefore important that the protocol is well suited for the target group and a simple protocol is considered important for minimizing error in plastic related citizen science projects^47^. In the mass experiment, the thoroughly developed protocol ensured that all participants had the ability to collect and categorize the data, supervised by the responsible teacher. Danish school classes contained 21.2 students on average in 2019. This number have been relatively constant over the past 5 years (21.2–21.3).

### Descriptive data presentation and analytic statistics

All data are expressed as total number of litter items for each category across all nature types, as well as for each nature type individually. In order to analyze differences in concentrations of plastic items between the nature types, plastic items per m^2^ were calculated using the individual transects length and width. Difference in central tendencies were estimated in three different ways; as median ± median absolute deviation (MAD), mean ± standard deviation (SD) and trimmed mean (10%) ± standard deviation (SD)^48^. All three way of reporting central tendencies are described by Joint Research Center (JRC) in their Marine Litter baseline report^48^. Median is recommended in the report but presentation of mean and trimmed mean (10%) provides an indication of the distribution of data around the central value. (Trimmed mean (10%) is a method of averaging that removes a 10% of the largest and smallest values before calculating the mean, thus neglecting the extreme values of the dataset). Since data were generally not normally distributed (supplementary material 2), Kruskal–Wallis rank sum test followed by pairwise comparison using Wilcoxon rank sum test were used to assess significant differences between the median values (p-value: 0.05). Even though non-systematic errors were unlikely due to the large data set, we used boxplots to identify potential outliers^49,50^ (Supplemental material 2). Participants that had reported potential outliers were contacted in order to clarify whether the data were reported correct. Additionally, participants reporting suspicious locations, e.g. gps-coordinates for locations outside the Danish Realm or locations at sea, were contacted for verification of the sampling locations. Two hundred and eighty-one schools were contacted, first with a mail and then with a phone call, if they did not respond to the mail. Of the 281 contacted 120 responded. Out of these, nine confirmed incorrect data reporting. Data from the nine together with the those that never responded were omitted from the database. For non-suspicious sampling locations, random sampling was done to confirm correct gps-coordinates. Fifty random chosen participants were contacted with a similar approach to verify that positions (gps coordinates) and reported data were correct. All of these were confirmed by the participants. Data were stored at Cern’s repository (Zenodo) and can be accessed at https://doi.org/10.5281/zenodo.3886974.

Guidelines and protocols were made in the collaboration with Astra – the National center for Science education. Astra has mandate under the National Center for Science Education Act adopted in 2018. The Act provides legal basis for Astra to develop protocols for and execute sampling of data in relation to the Mass Experiment. The Minister of Education and the Minister of Science appoint board members of Astra individually, in order to ensure that the board has both legal and ethical competences in accordance with legal requirements. Astra Board ethics committee approved the study and all activities in the Mass Experiment. All procedures were carried out in accordance with the legal mandate of the National Center for Science Education Act^51^. Informed consent was obtained from all participants Informed consent was obtained from all participants. Protocols and collection of data were conducted in collaboration with Roskilde University, Department of Science and Environment. All data collected were anonymized upon collection in accordance with the General Data Protection Regulation.

## Supplementary information

Supplementary file1

## References

[1] Geyer R, Jambeck J, Law K (2017). Production, use, and fate of all plastics ever made. Sci. Adv..

[2] GESAMP. Sources, fate and effects of microplastics in the marine environment: a global assessment (ed. Kershaw, P. J.). (IMO/FAO/UNESCO-IOC/UNIDO/WMO/IAEA/UN/UNEP/UNDP Joint Group of Experts on the Scientific Aspects of Marine Environmental Protection). Rep. Stud. GESAMP No. 90, 96p. (2015).

[3] The future of plastic. *Nat. Commun.* **9**, 2157 (2018) 10.1038/s41467-018-04565-2 PMC5988835 29872038

[4] Steffen W (2015). Planetary boundaries: guiding human development on a changing planet. Science.

[5] Li WC, Tse HF, Fok L (2016). Plastic waste in the marine environment: a review of sources, occurrence and effects. Sci. Total Environ..

[6] Jambeck JR (2015). Plastic waste inputs from land into the ocean. Science.

[7] SAPEA (2019). A Scientific Perspective on Microplastics in Nature and Society.

[8] Haward M (2018). Plastic pollution of the world’s seas and oceans as a contemporary challenge in ocean governance. Nat. Commun..

[9] Syberg K, Hansen SF, Christensen TB, Khan FR (2018). Risk perception of plastic pollution: Importance of stakeholder involvement and citizen science. Handb. Environ. Chem..

[10] UNEP. *Single-Use Plastic: A Roadmap for Sustainability*. *United Nation Environment Programme* (2018).

[11] Nelms SE (2017). Marine anthropogenic litter on British beaches: a 10-year nationwide assessment using citizen science data. Sci. Total Environ..

[12] Rosevelt C, Los Huertos M, Garza C, Nevins HM (2013). Marine debris in central California: Quantifying type and abundance of beach litter in Monterey Bay, CA. Mar. Pollut. Bull..

[13] Mayoma BS, Mjumira IS, Efudala A, Syberg K, Khan FR (2019). Collection of anthropogenic litter from the shores of Lake Malawi: characterization of plastic debris and the implications of public involvement in the African Great Lakes. Toxics.

[14] Howard, B. C., Gibbens, S., Zachos, E. & Parker, L. A running list of action on plastic pollution. National Geographic. https://www.nationalgeographic.com/environment/2018/07/ocean-plastic-pollution-solutions/ (2019).

[15] Ocean Conservacy. International Coastal Cleanup. https://oceanconservancy.org/trash-free-seas/international-coastal-cleanup/ (2019).

[16] Haarr ML, Pantalos M, Hartviksen MK, Gressetvold M (2020). Citizen science data indicate a reduction in beach litter in the Lofoten archipelago in the Norwegian Sea. Mar. Pollut. Bull..

[17] EEA. Marine LitterWatch. Citizens collect plastic and data to protect Europe’s marine environment. European Environmental Agency, Copenhagen. https://www.eea.europa.eu/publications/marine-litter-watch (2018).

[18] GESAMP. Guidelines or the monitoring and assessment of plastic litter and microplastics in the ocean (eds Kershaw P. J., *et al.*), (IMO/FAO/UNESCO-IOC/UNIDO/WMO/IAEA/UN/UNEP/UNDP/ISA Joint Group of Experts on the Scientific Aspects of Marine Environmental Protection). Rep. Stud. GESAMP No. 99, 130p. (2019).

[19] Huerta Lwanga E (2017). Field evidence for transfer of plastic debris along a terrestrial food chain. Sci. Rep..

[20] Malizia A, Monmany-Garzia AC (2019). Terrestrial ecologists should stop ignoring plastic pollution in the Anthropocene time. Sci. Total Environ..

[21] Kiessling T (2019). Plastic Pirates sample litter at rivers in Germany: Riverside litter and litter sources estimated by schoolchildren. Environ. Pollut..

[22] Tobin consulting. *Monitoring System Litter Monitoring Body System Results 2018 Prepared for :* (2018).

[23] Naturstyrelsen. Vejledning om naturbeskyttelsesloven. *Report* (2008). Available at: https://naturstyrelsen.dk/publikationer/2008/dec/vejledning-om-naturbeskyttelsesloven/. (Accessed: 30th April 2002)

[24] Hartley BL (2018). Exploring public views on marine litter in Europe: Perceived causes, consequences and pathways to change. Mar. Pollut. Bull..

[25] Nature, D. council for. affaldsindsamling 2019. (2019). Available at: https://www.affaldsindsamlingen.dk/arets-resultater/. (Accessed: 21st April 2020)

[26] Rath JM, Rubenstein RA, Curry LE, Shank SE, Cartwright JC (2012). Cigarette litter: Smokers attitudes and behaviors. Int. J. Environ. Res. Public Health.

[27] Feld, L., Metcalfe, A. & Strand, J. National monitoring of beach litter in Denmark. Amounts and composition of marine litter on reference beaches. DCE - Danish Centre for Environment and Energy. https://dce.au.dk/fileadmin/dce.au.dk/Udgivelser/Notater_2018/Beach_litter_at_Danish_reference_beaches_2018.pdf (2018).

[28] Marine Litter MSFD Technical Subgroup (2010). Guidance on Monitoring of Marine Litter in European Seas. Ref. Rep. Joint Res. Centre Eur. Commiss..

[29] EC. *Directive (EU) 2019/904 of the european parliament and of the council of 5 June 2019 on the reduction of the impact of certain plastic products on the environment*. **2019**, 1–19 (2019).

[30] European Commission. Commission Staff Working Document Impact Assessment Accompanying the document Proposal for a Directive of the European Parliament and of the Council on the reduction of the impact of certain products on the environment. **SWD(2018)**, 76 (2018).

[31] Danish EPA. From land filling to recovery – Danish waste management from the 1970s until today. The Danish action plan for promotion of eco-efficient technologies – Danish Lessons. Danish Ministry of the Environment, Environmental Protection agency. https://eng.ecoinnovation.dk/media/mst/8051407/Affald_Baggrundsartikel_affald_web_15.01.13.pdf (2013).

[32] EC. Directive (EU) 2015/720 of the European Parliament and of the Council of 29 April 2015 amending Directive 94/62/EC as regards reducing the consumption of lightweight plastic carrier bags. *Off. J. Eur. Union* **2014**, 20–30 (2015).

[33] Anastasio, M. & Nix, J. *Plastic Bag Levy in Ireland*. (2016).

[34] UN Secretariat, D. for S. D., Affairs, D. of E. and S. & Building. *Transforming our world: the 2030 Agenda for sustainable development*. *Transforming our world: the 2030 Agenda for sustainable development* (2016). 10.1201/b20466-7

[35] Fritz S (2019). Citizen science and the United Nations Sustainable Development Goals. Nat. Sustain..

[36] Sonne C, Alstrup AKO (2019). Using citizen science to speed up plastic collection and mapping of urban noise: Lessons learned from Denmark. Mar. Pollut. Bull..

[37] Damerell P, Howe C, Milner-Gulland EJ (2013). Child-orientated environmental education influences adult knowledge and household behaviour. Environ. Res. Lett..

[38] OECD. *OECD Reviews of School Resources: Denmark 2016*. (2016). 10.1787/9789264265530-5-en

[39] Statistics Denmark. Population at the first day of the quarter by time, age and sex. Available at: https://www.statbank.dk/statbank5a/selectvarval/saveselections.asp.

[40] Føroya landsstýri. Facts and Figures. (2019). Available at: https://www.faroeislands.fo/the-big-picture/facts-and-figures/. (Accessed: 24th March 2020)

[41] Statistics Greenland. *Greenland in Figures Greenland*. *Statistics Greenland* (2013). 978-87-998113-0-4

[42] Kosmala M, Wiggins A, Swanson A, Simmons B (2016). Assessing data quality in citizen science. Front. Ecol. Environ..

[43] Bird TJ (2014). Statistical solutions for error and bias in global citizen science datasets. Biol. Conserv..

[44] Galgani F, Hanke G, Werner S, Vrees L. De (2013). Marine litter within the European Marine Strategy Framework Directive. Mar. Sci..

[45] Astra. Masseeksperimentet 2019. (2019). Available at: https://naturvidenskabsfestival.dk/tildinundervisning/masseeksperiment-2019-plastforurening-i-vand.

[46] NELSON, E. *Radically Elementary Probability Theory. (AM-117)*. (Princeton University Press, Princeton, 1987). 10.2307/j.ctt1b7x772

[47] Rambonnet L, Vink SC, Land-Zandstra AM, Bosker T (2019). Making citizen science count: Best practices and challenges of citizen science projects on plastics in aquatic environments. Mar. Pollut. Bull..

[48] del Mar Chaves Montero, M. *et al.*. EU Marine Beach Litter Baselines. Analysis of the pan-European 2012-2016 beach litter dataset. MSFD technical group on Marine litter, Joint Research Center. 10.2760/16903 (2019).

[49] Sokal RR, Rohlf FJ (1981). Biometry The Principles and Practice of Statistics in Biological Research.

[50] Zuur AF (2009). Mixed effects models and extensions in ecology with R.

[51] LOV_nr_1320_af_27/11/2018. *Lov om et nationalt naturfagscenter*. **2018**, 11–13 (2020).

