# Supplementary material for: A nationwide assessment of plastic pollution in the Danish realm using citizen science
Source: Sci Rep. 2020 Oct 20;10:17773. doi: 10.1038/s41598-020-74768-5 (PMC7576202; doi:10.1038/s41598-020-74768-5)
Supplement: Supplementary file 1 — Supplementary file1 [file 41598_2020_74768_MOESM1_ESM.pdf]

Supplementary information

A nationwide assessment of plastic pollution in the Danish realm using citizen science

Kristian Syberg[1]*, Annemette Palmqvist[1], Farhan R. Khan[1], Jakob Strand[2], Jes Vollertsen[3], Lauge Peter Westergaard Clausen[4], Louise Feld[2], Nanna B. Hartmann[4], Nikoline Oturai[1], Søren Møller[1], Torkel Gissel Nielsen[5], Yvonne Shashoua[6] and Steffen Foss Hansen[4]

1 Roskilde University, Department of Science and Environment, Denmark

2 Aarhus University, Department of Bioscience, Denmark

3 Aalborg University, Department of Civil Engineering, Denmark

4 Technical University of Denmark, Department of Environmental Engineering, Denmark

5 Technical University of Denmark, National Institute of Aquatic resources, Denmark

6 National museum of Denmark, Environmental Archaeology and Materials Science, Denmark

* E-mail contact: ksyberg@ruc.dk

Supplementary information. Annex I - Plastic pieces collected in the 22 categories distributed on the 8 nature types

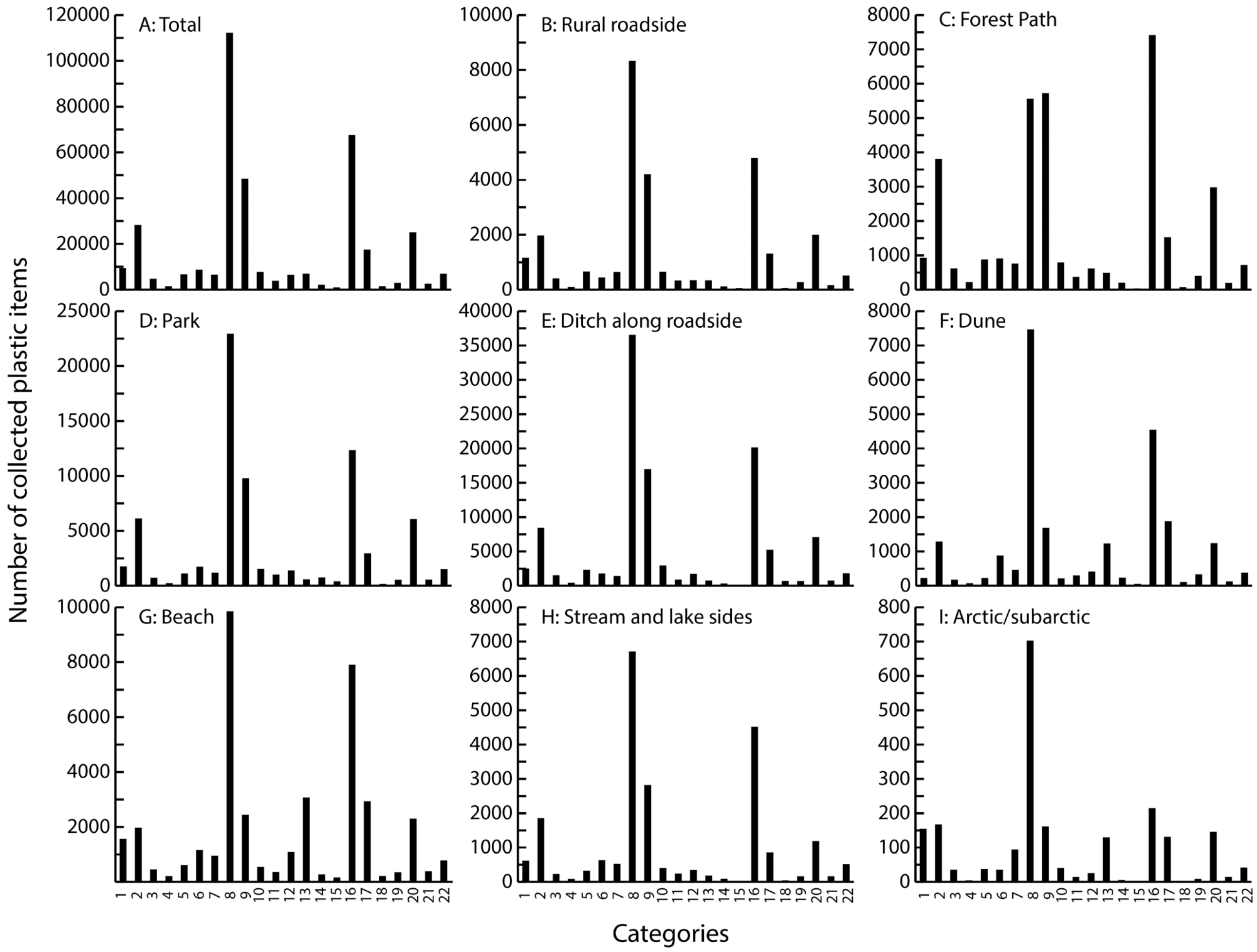

Number of plastic items distributed along the 22 plastic categories for the total dataset (A) as well as each of the eight nature types (B-I). The numbers correspond to 1. Shopping bags, 2. Small plastic bags, 3. Drink bottles (≤0.5l), 4. Drink bottles (>0.5l), 5. Food containers incl. fast food containers, 6. Plastic caps/lids; drinks, 7. Plastic caps/lids; unidentified, 8. Cigarette butts and filters, 9. Crisp packets/sweet wrappers, 10. Cups and cup lids, 11. Cutlery and trays, 12. Straws and stirrers, 13. String and cord, 14. Nets and pieces of net < 50 cm, 15. Nets and pieces of net > 50 cm, 16. Plastic pieces 2.5 cm > < 50cm, 17. Polystyrene pieces 2.5 cm > < 50cm, 18. Cotton bud sticks, 19. Sanitary towels/panty liners/backing strips, 20. Other plastic/polystyrene items (identifiable), 21. Balloons and balloon strings and sticks, 22. Other rubber pieces.

Supplementary material. Annex II – statistical assessments

Distribution of samples based on densities of plastic items (top) and frequency (bottom)

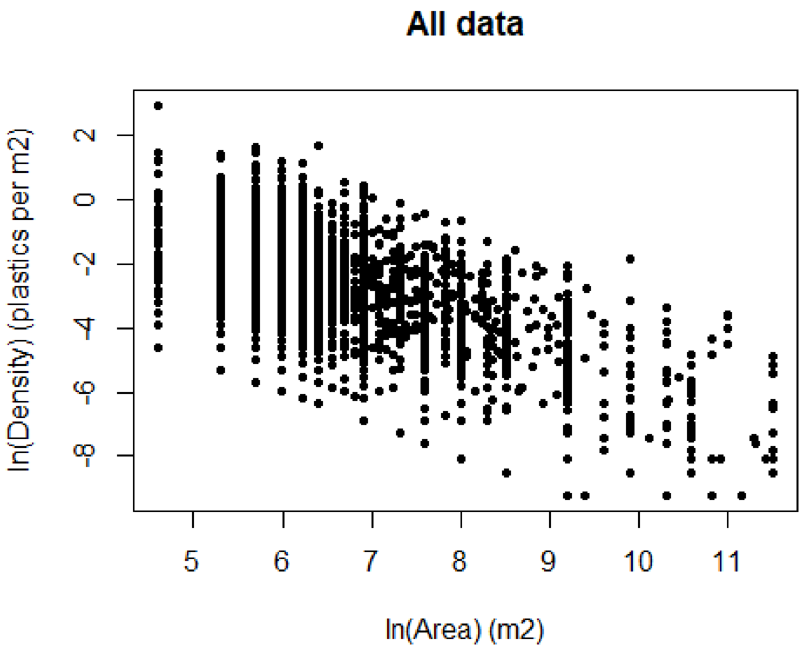

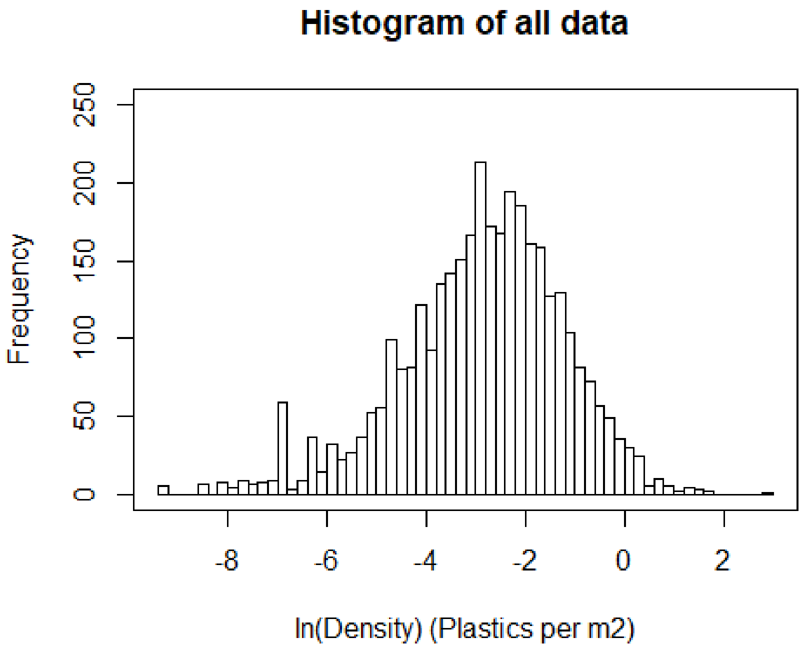

Data distribution in the eight different nature types. Histograms and box plots on linear and logarithmic scales.

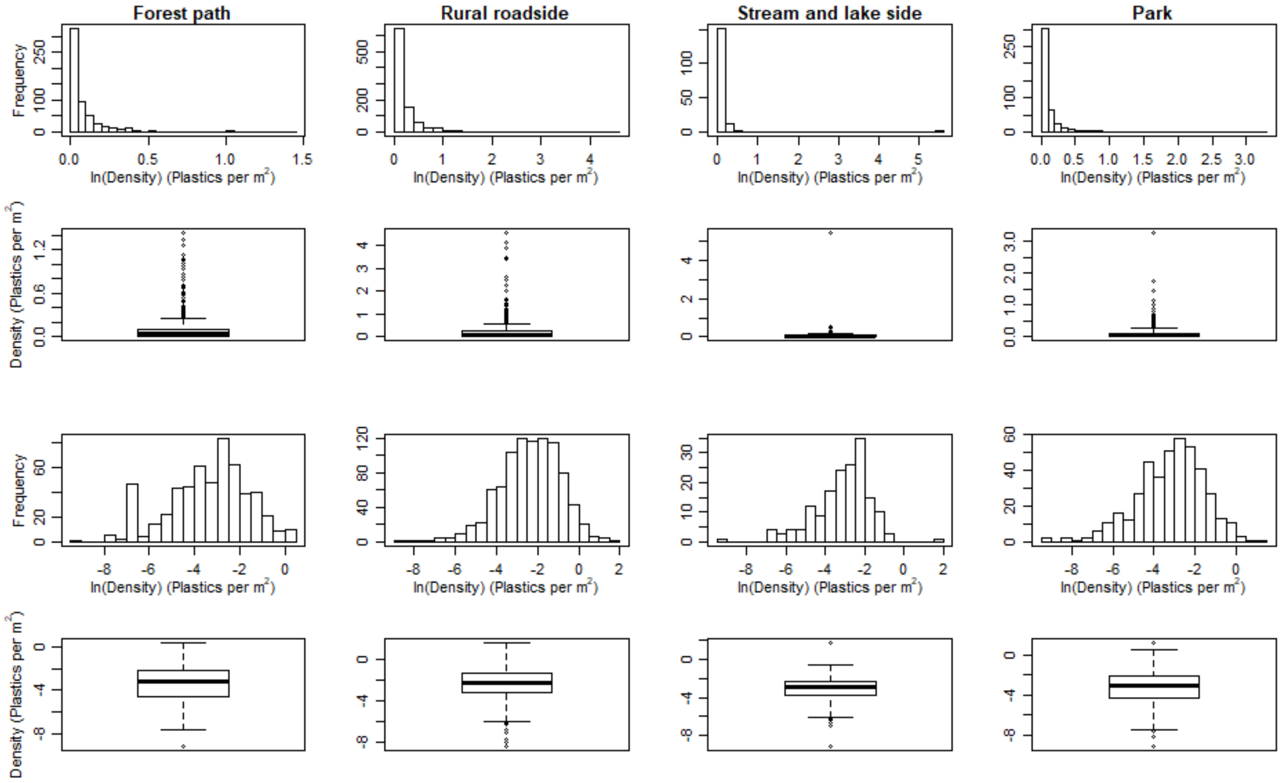

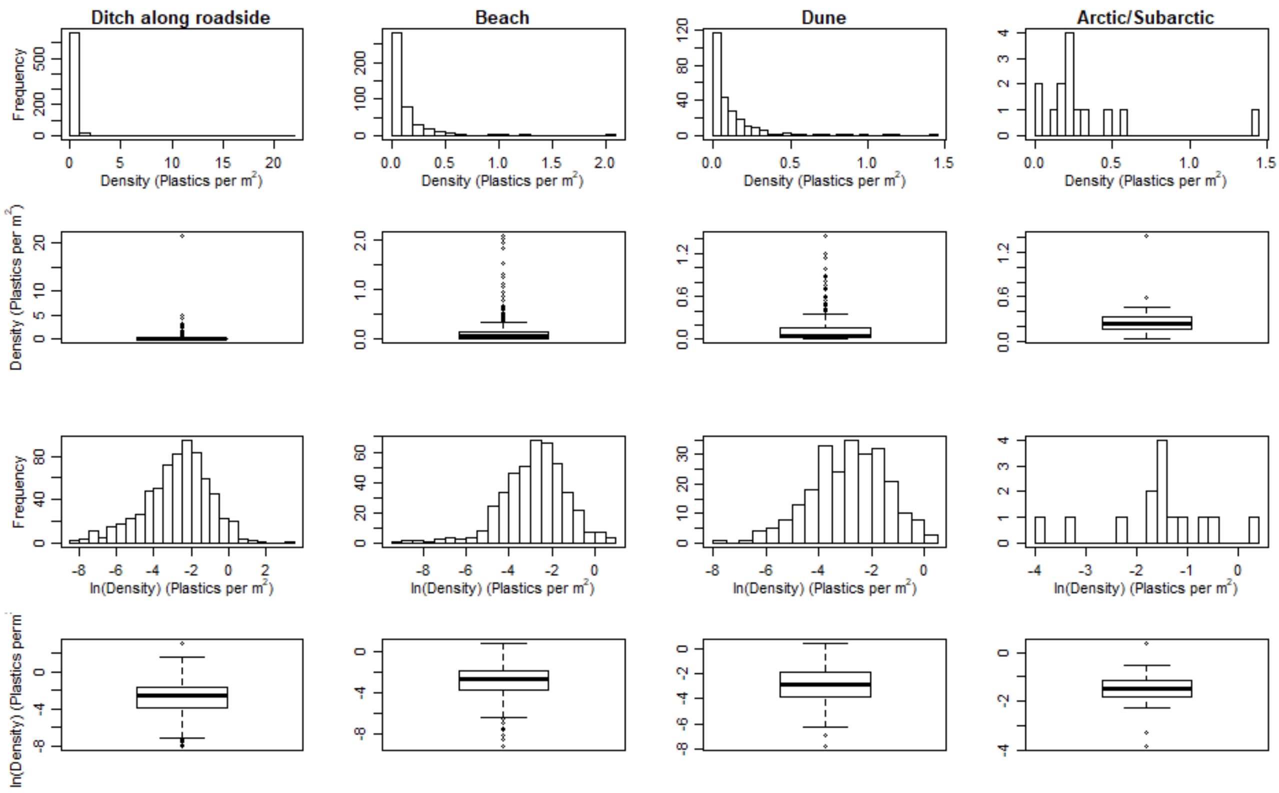

Data distribution in the 22 different plastic categories. Histograms and box plots on linear and logarithmic scales.

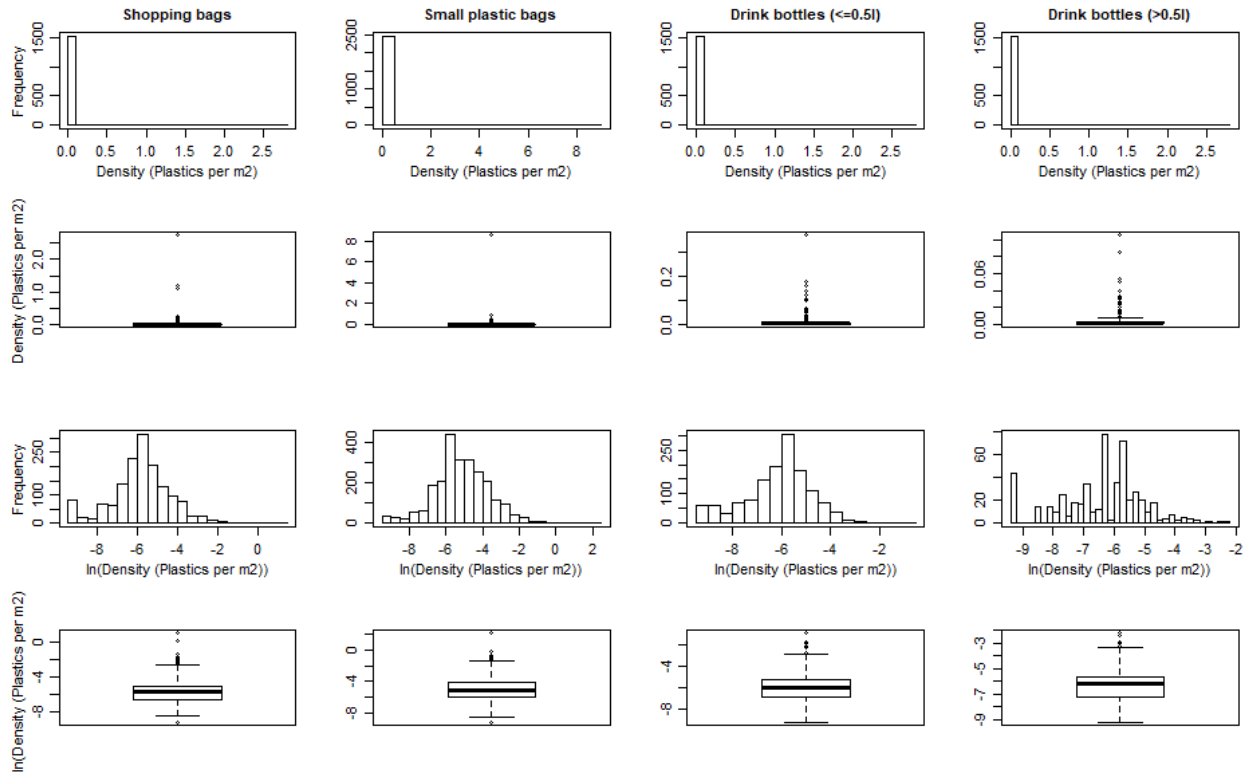

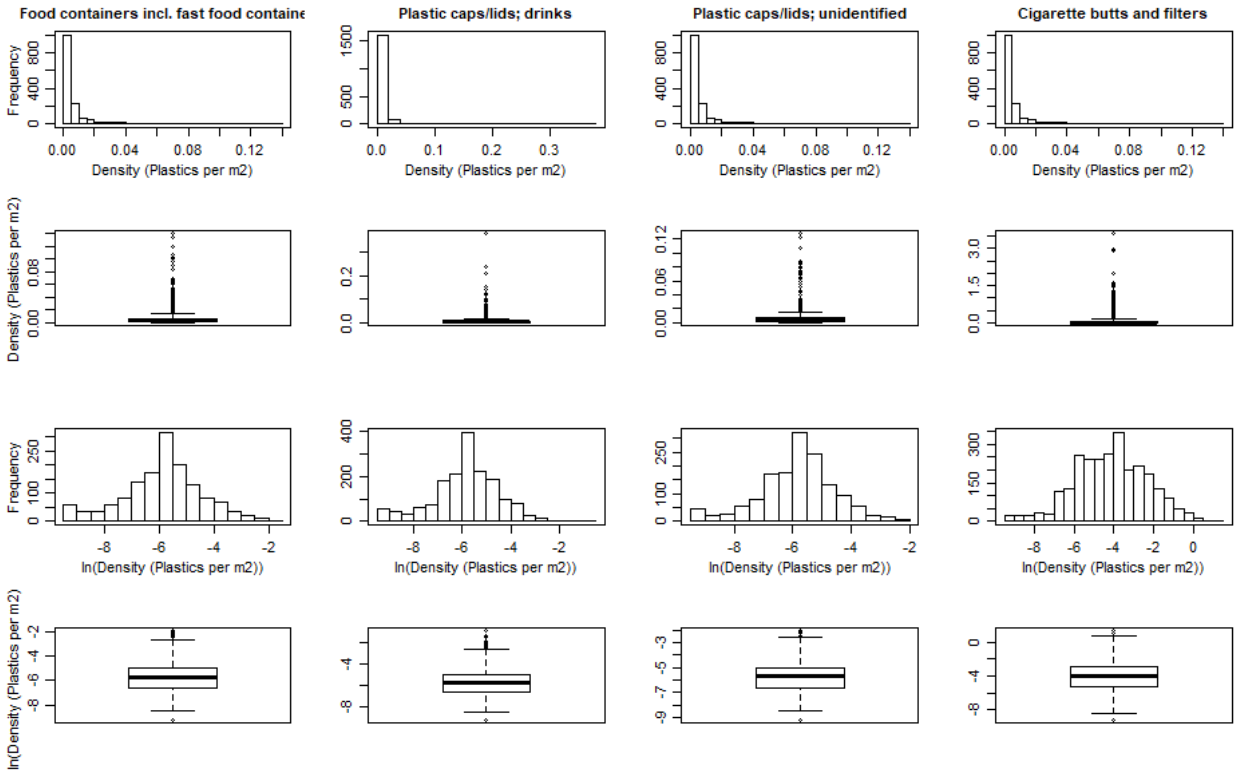
Food containers incl. fast food containe
Plastic caps/lids; drinks
Plastic caps/lids; unidentified
Cigarette butts and filters
Frequency
Density (Plastics per m2)
ln(Density (Plastics per m2))

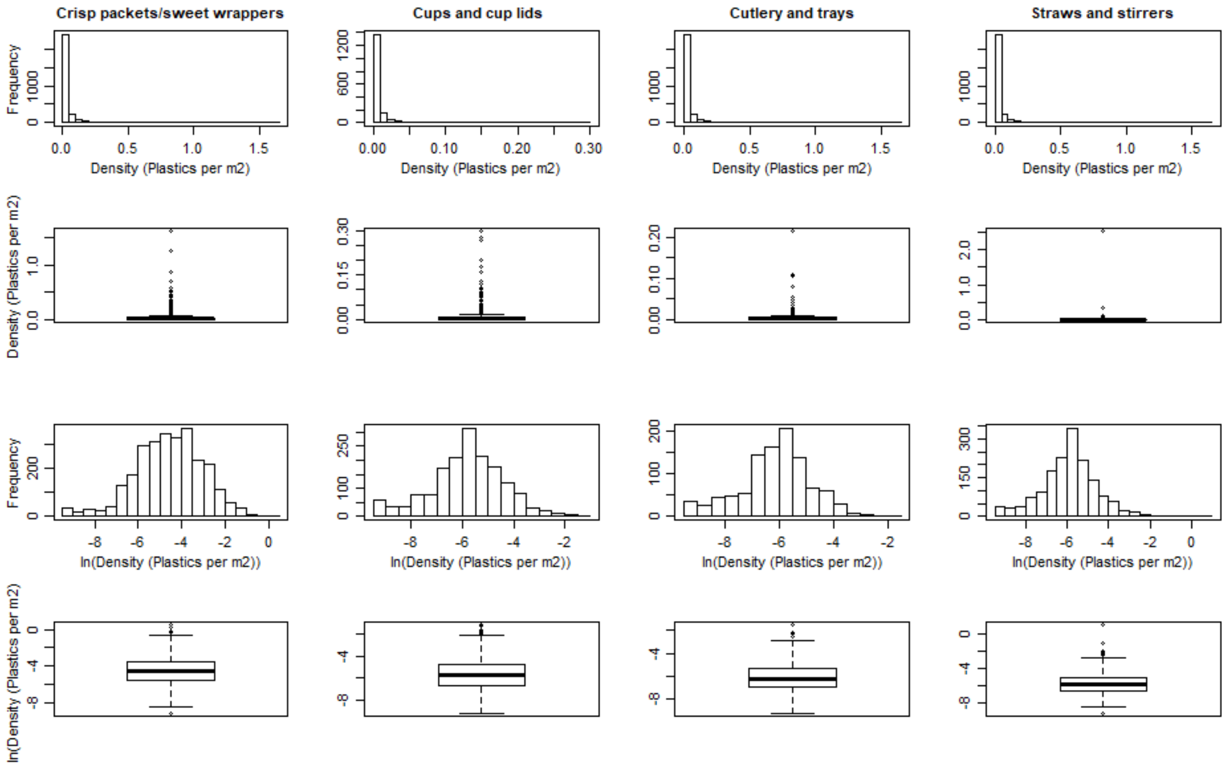
Crisp packets/sweet wrappers
Cups and cup lids
Cutlery and trays
Straws and stirrers
Frequency
Density (Plastics per m2)
ln(Density (Plastics per m2))

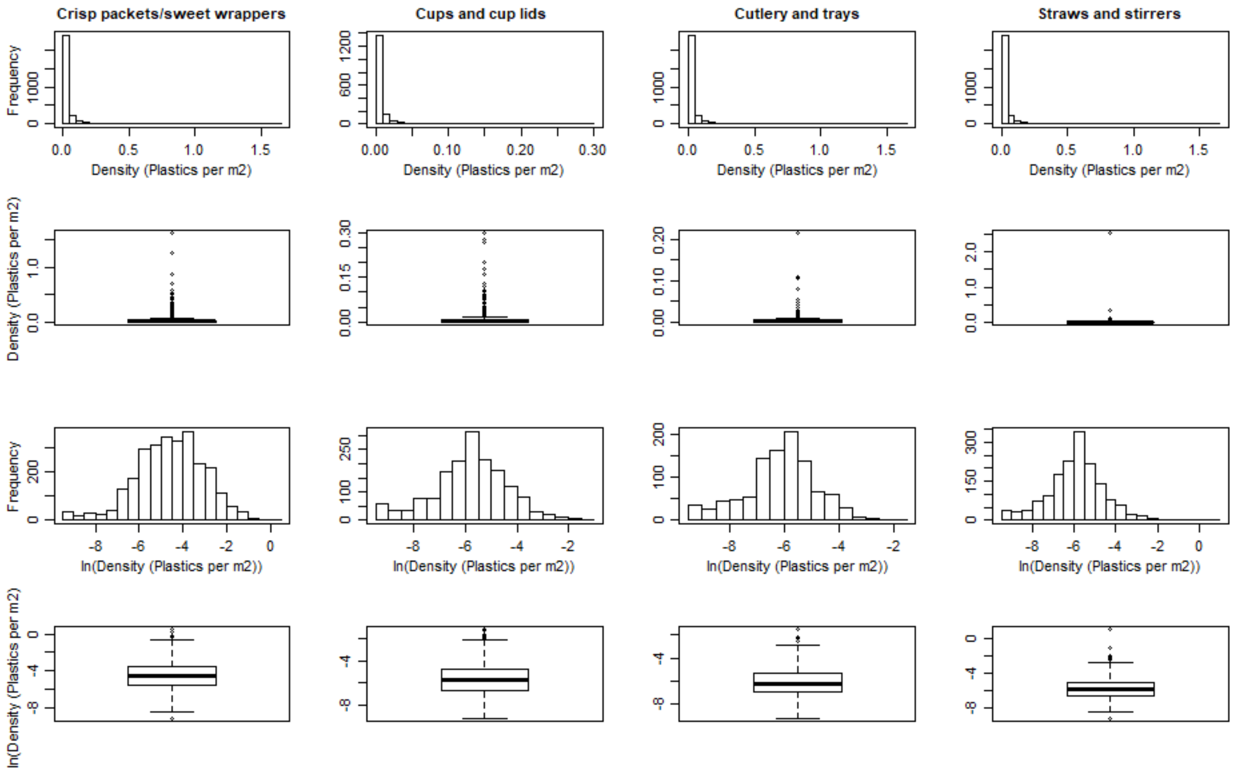
Crisp packets/sweet wrappers
Cups and cup lids
Cutlery and trays
Straws and stirrers
Frequency
Density (Plastics per m2)
ln(Density (Plastics per m2))

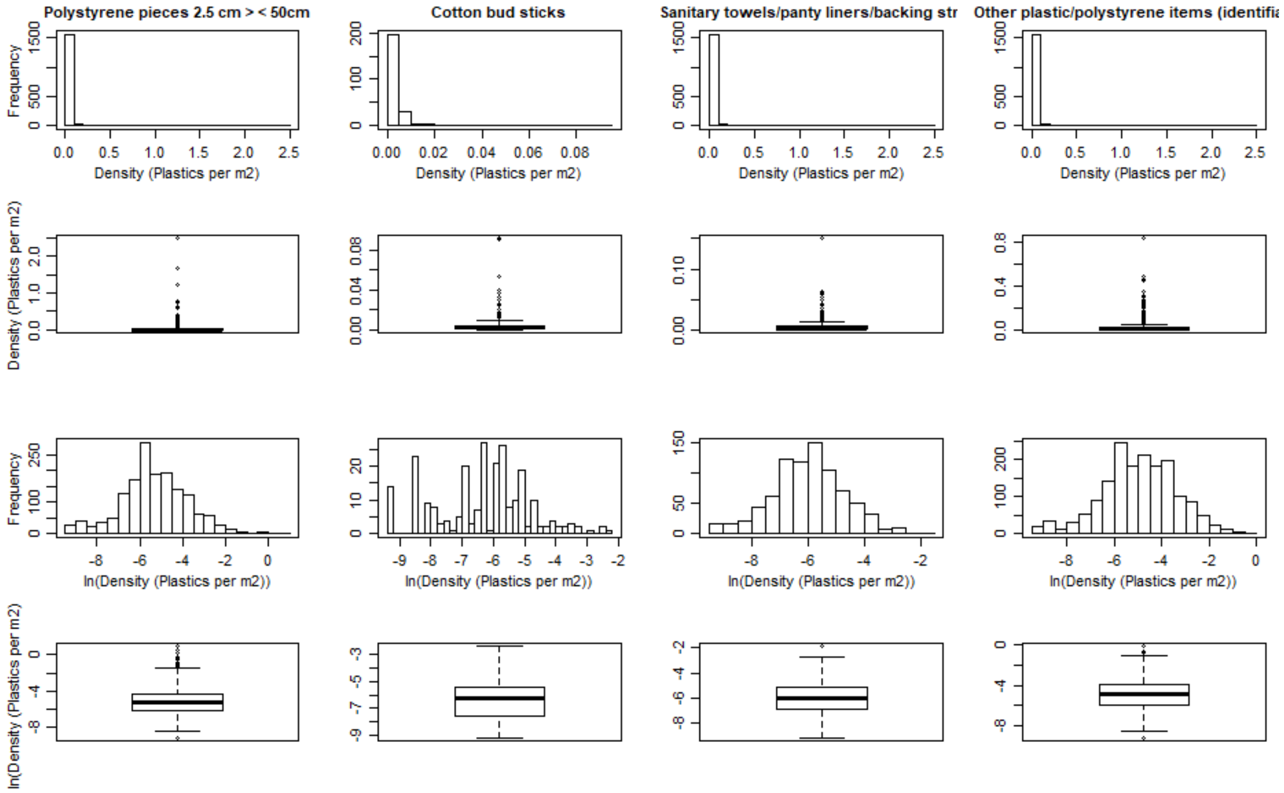
Polystyrene pieces 2.5 cm > < 50cm
Cotton bud sticks
Sanitary towels/panty liners/backing str
Other plastic/polystyrene items (identifia
Frequency
Density (Plastics per m2)
ln(Density (Plastics per m2))

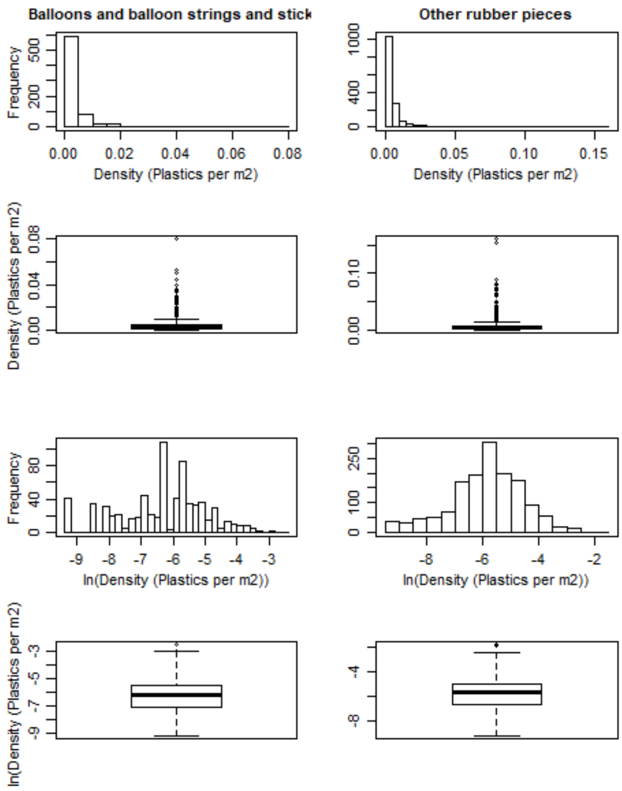

Balloons and balloon strings and stick
Other rubber pieces
Frequency
Density (Plastics per m2)
ln(Density (Plastics per m2))

Supplementary materials Annex III – Examples of teaching materials

i) “Table cloth” with pictograms of the 22 categories of plastic for sorting and counting the samples in the field (In Danish).

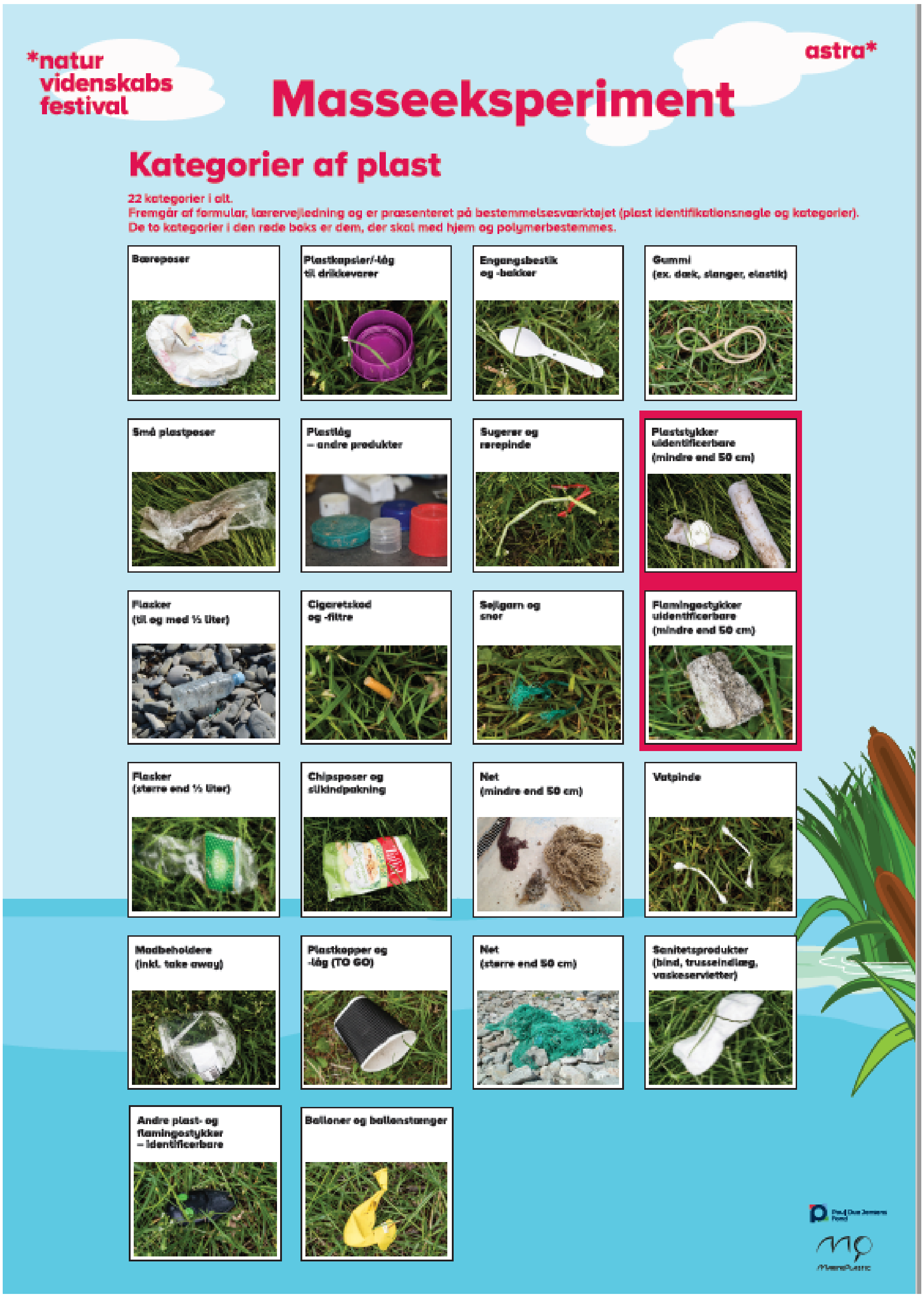

ii) The flow of plastic through society and into the environment. Illustration provided in the teacher version of the protocol, to facilitate teaching about plastic pollution in relation to participating in the Mass Experiment (in Danish).

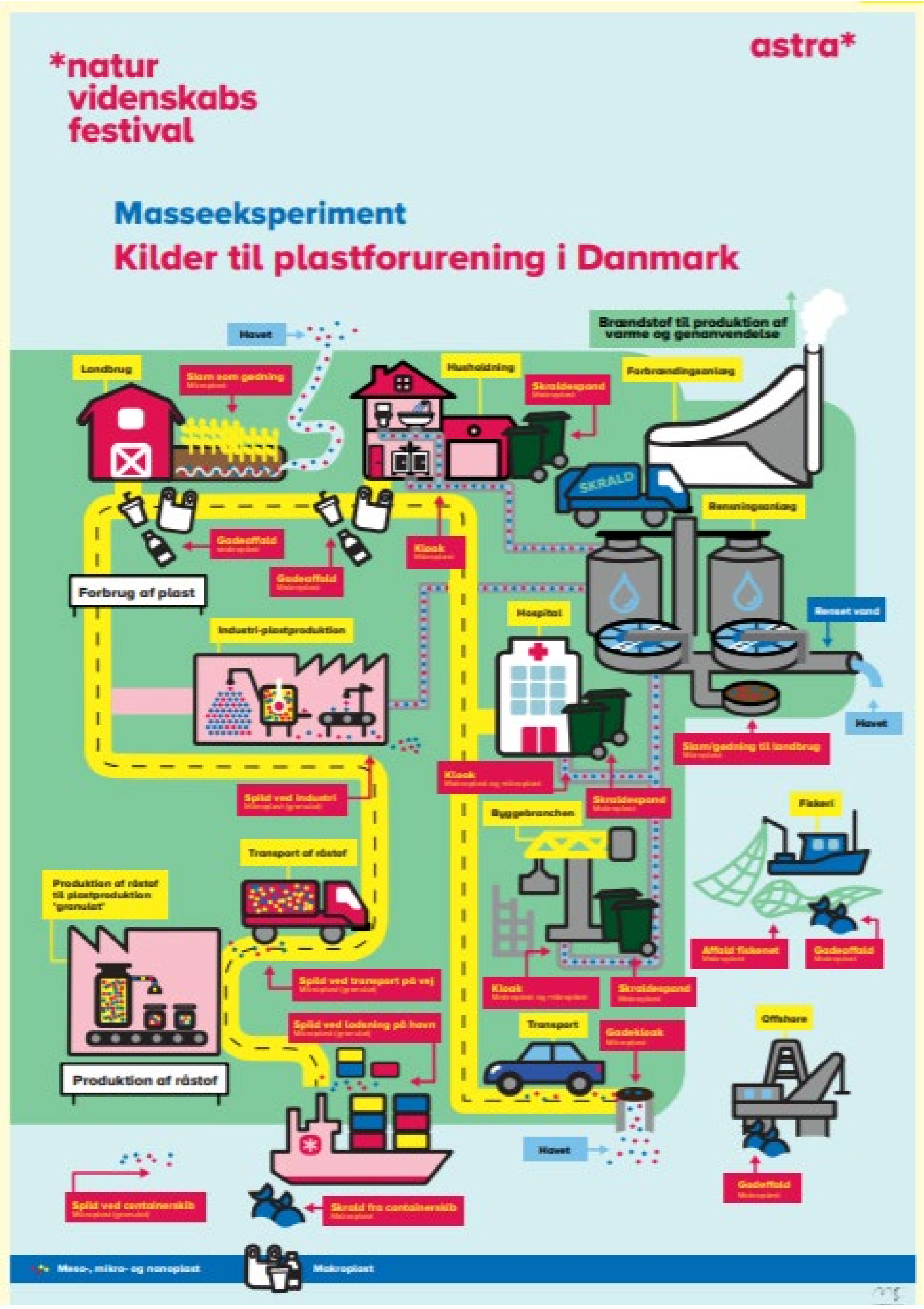

iii) Screenshots from the database interface that the participants used to report the samples in. Above: overview of the different categories and information that participants could report. Below: Screenshot from the database used to collect, sort and analyze the data.

Indsendelser Analyse Tabel Hent Ryd

Showing 1 - 50 of 3564. Show 10 | 50 | 100 | 200 | 500 | 1000 | Alle results per page.

#	INDSENDT	BRUGER	IP-ADRESSE	HOLD / KLASSEID	HVILKEN NATURTYPE HAR I INDSAMLET PLAST FRA?	NAVN (ELLER ADRESSE) PÅ OMRÅDET	GPS-KOORDINATER	OMRÅDETS BREDDE	OMRÅDETS LÆNGDE	HVORNÅR FORETOG I INDSAMLINGEN?	G3. BÆREPOSER	G4. SMÅ PLASTPOSER	G7. FLASKER (TIL OG MED 1/2 LITER)	G8. FLASKER (STØRRE END 1/2 LITER)	G10. MADBEHOLDERE (INKL. FASTFOOD)	G21. PLASTKAPSLER/-LÅG TIL DRIKKEVARER	G23. PLASTLÅG - ANDRE PRODUKTER	G27. CIGARETSKOD OG -FILTRE	G30. CHIPSPOSER OG SLIKINDPAKNING	G33. PLASTKOPPER OG -LÅG (TO GO)	G34. ENGANGSBESTIK OG -BAKKER	G35. SUGERØR OG RØREPINDE	G50. SEJLGARN OG SNOR	G53. NET (MINDRE END 50 CM)	G54. NET (STØRRE END 50 CM)	G79. PLASTSTYKKER UIDENTIFICERBARE (MINDRE END 50 CM)	G82. FLAMINGOSTYKKER UIDENTIFICERBA (MINDRE END 50 CM)
3567	01. nov. 2019	Anonymous (ikke efterprøvet)	185.85.44.153	280327-2037-3	Parkområde	Birkerød Station	55.84057, 12.42196	7	100	01. nov	1	10	0	0	2	0	0	0	19	0	0	1	1	0	0	8	1
3566	01. nov. 2019	Anonymous (ikke efterprøvet)	185.85.44.153	280327-2037-3	Skovstier	Birkerød Parkvej	55.84189, 12.41452	10	100	01. nov	0	30	6	1	3	4	1	0	27	10	0	3	0	1	0	15	0
3565	31. okt. 2019	Anonymous (ikke efterprøvet)	77.233.233.119	735003-2980-1	Parkområde	Skolestien, Ørsted	56.3027, 10.2009	5	100	08. okt	12	34	5	0	3	1	11	27	19	0	0	1	0	0	0	24	2
3564	27. okt. 2019	Anonymous (ikke efterprøvet)	93.167.48.153	101049-3005-2	Parkområde	Amager Fælled, Ørestad Boulevard, 2300 Kbh. S.	55.647193, 12.585485	8	100	26. sep	0	4	1	0	0	2	4	28	6	0	0	1	3	0	0	3	0

6500 MX - resultater data til studio 2019 110919

Fil Rediger Se Indsæt Formatér Data Værktøjer Tilføjelser Hjælp Sidste redigering fandt sted for få sekunder siden

Del

100% kr % .0 .00 123 Standard (... 10

31

A	B	C	D	E	F	G	H	I	J	K	L	
erienummer	SID	Submitted Time	Completed Time	Modified Time	Kladde	IP-adresse	UID	Brugernavn	Hold / KlasseID	Hvilken naturty	Navn (eller adresse) på område	GPS-ko
27	278551	29-08-2019 14:30	29-08-2019 14:30	29-08-2019 14:30	0	94.101.218.10	6	bbc@astra.dk	101062-1804-1	Opskylningszone p	TESTnavn	55.7217
28	278556	29-08-2019 14:31	29-08-2019 14:31	29-08-2019 14:31	0	94.101.218.10	6	bbc@astra.dk	101062-1804-1	Åbred (søbred, va	testnavn2	55.7217
29	278561	29-08-2019 14:32	29-08-2019 14:32	29-08-2019 14:32	0	94.101.218.10	6	bbc@astra.dk	101062-1804-1	Vejkant langs mar	test3	55.7217
30	280021	11-09-2019 11:17	11-09-2019 11:17	11-09-2019 11:17	0	78.156.102.169	0		613008-1847-1	Grøftekant langs v	Bøgballe, Fælledvej	55.8261
31	280026	11-09-2019 11:23	11-09-2019 11:23	11-09-2019 11:23	0	78.156.102.169	0		613008-1847-1	Åbred (søbred, va	Bøgballe vej	55.8236
32	280031	11-09-2019 11:26	11-09-2019 11:26	11-09-2019 11:26	0	78.156.102.169	0		613008-1847-1	Vejkant langs mar	Bøgballe	55.8279
33	280036	11-09-2019 11:35	11-09-2019 11:35	11-09-2019 11:35	0	5.186.119.40	0		ID fra tilmelding -	Grøftekant langs v	Nr. Alslev	54.8946
34	280041	11-09-2019 11:35	11-09-2019 11:35	11-09-2019 11:35	0	5.186.119.40	0		ID fra tilmelding -	Grøftekant langs v	4840 Nørre Alslev	54.8943
35	280046	11-09-2019 11:43	11-09-2019 11:43	11-09-2019 11:43	0	5.186.119.40	0		ID fra tilmelding -	Vejkant langs mar	Falkerslev	54.8393
36	280051	11-09-2019 11:47	11-09-2019 11:47	11-09-2019 11:47	0	5.186.119.40	0		ID fra tilmelding -	Parkområde	stubbekøbing	54.8911
37	280056	11-09-2019 11:47	11-09-2019 11:47	11-09-2019 11:47	0	5.186.119.40	0		ID fra tilmelding -	Parkområde	Lillegade 17	54.8243
38	280061	11-09-2019 11:47	11-09-2019 11:47	11-09-2019 11:47	0	5.186.119.40	0		ID fra tilmelding -	Parkområde	Nr. Alslev	54.8984
39	280066	11-09-2019 11:48	11-09-2019 11:48	11-09-2019 11:48	0	5.186.119.40	0		ID fra tilmelding -	Grøftekant langs v	Sortsø	54.9228
40	280071	11-09-2019 11:49	11-09-2019 11:49	11-09-2019 11:49	0	5.186.119.40	0		ID fra tilmelding -	Parkområde	Stubbekøbing	54.8890
41	280076	11-09-2019 11:51	11-09-2019 11:51	11-09-2019 11:51	0	5.186.119.40	0		ID fra tilmelding -	Vejkant langs mar	stubbekøbingvej 4840	54.8975
42	280081	11-09-2019 12:05	11-09-2019 12:05	11-09-2019 12:05	0	5.179.88.34	0		461008-1896-1	Parkområde	Odense å	55.3907
43	280086	11-09-2019 12:20	11-09-2019 12:20	11-09-2019 12:20	0	212.98.72.247	0		761006-2971-1	Parkområde	Lunden, Rødkærsbro	56.3520
44	280091	11-09-2019 12:54	11-09-2019 12:54	11-09-2019 12:54	0	78.156.102.169	0		613008-1847-1	Grøftekant langs v	Fælledvej	55.8224
45	280096	11-09-2019 12:56	11-09-2019 12:56	11-09-2019 12:56	0	78.156.102.169	0		613008-1847-1	Grøftekant langs v	bøgballe	55.8225
46	280101	11-09-2019 13:04	11-09-2019 13:04	11-09-2019 13:04	0	78.156.102.169	0		613008-1847-1	Grøftekant langs v	bøballe Friskole	55.8225
47	280106	11-09-2019 13:15	11-09-2019 13:15	11-09-2019 13:15	0	78.156.102.169	0		613008-1847-1	Vejkant langs mar	Bøgballe	55.8207
48	280111	11-09-2019 13:33	11-09-2019 13:33	11-09-2019 13:33	0	5.179.88.34	0		461008-1896-1	Åbred (søbred, va	Odense å	55.3894
49	280116	11-09-2019 13:40	11-09-2019 13:40	11-09-2019 13:40	0	5.179.88.34	0		461008-1896-1	Parkområde	Erik Bøghs Sti, Odense	55.3729
50	280121	11-09-2019 13:46	11-09-2019 13:46	11-09-2019 13:46	0	5.179.88.34	0		461008-1896-1	Åbred (søbred, va	Ådalen, Odense	55.3846
51	280126	11-09-2019 13:54	11-09-2019 13:54	11-09-2019 13:54	0	5.179.88.34	0		461008-1896-1	Parkområde	Odense å	55.3833
52	280141	11-09-2019 14:05	11-09-2019 14:05	11-09-2019 14:05	0	5.179.88.34	0		461008-1896-1	Parkområde	Munke Mose, Odense	55.3905
53	280156	11-09-2019 19:31	11-09-2019 19:31	11-09-2019 19:31	0	185.255.156.35	0		605300-2588-1	Parkområde	Den grønne legeplads bag ved Dagli	55.7054
54	280181	12-09-2019 08:07	12-09-2019 08:07	12-09-2019 08:07	0	131.165.203.187	0		367001-1903-2	Parkområde	Cykelsti i grønt område Nakskov.	54.8392
55	280196	12-09-2019 11:20	12-09-2019 11:20	12-09-2019 11:20	0	85.236.64.188	0		169001-2326-1	Skovstier	fløng 2640	55.6619
56	280201	12-09-2019 11:21	12-09-2019 11:21	12-09-2019 11:21	0	85.236.64.188	0		169001-2326-1	Grøftekant langs v	Marbjerg byvejen	55.6616
57	280251	12-09-2019 13:07	12-09-2019 13:07	12-09-2019 13:07	0	185.19.133.10	0		561401-3267-1	Skovstier	Den hemmelige skov	55.4954
58	280256	12-09-2019 13:07	12-09-2019 13:07	12-09-2019 13:07	0	185.19.132.196	0		561401-3267-1	Opskylningszone r	Mennesket ved Havet	55.4877

Supplementary information. Annex IV – Overview of outreach impact of the Mass Experiment

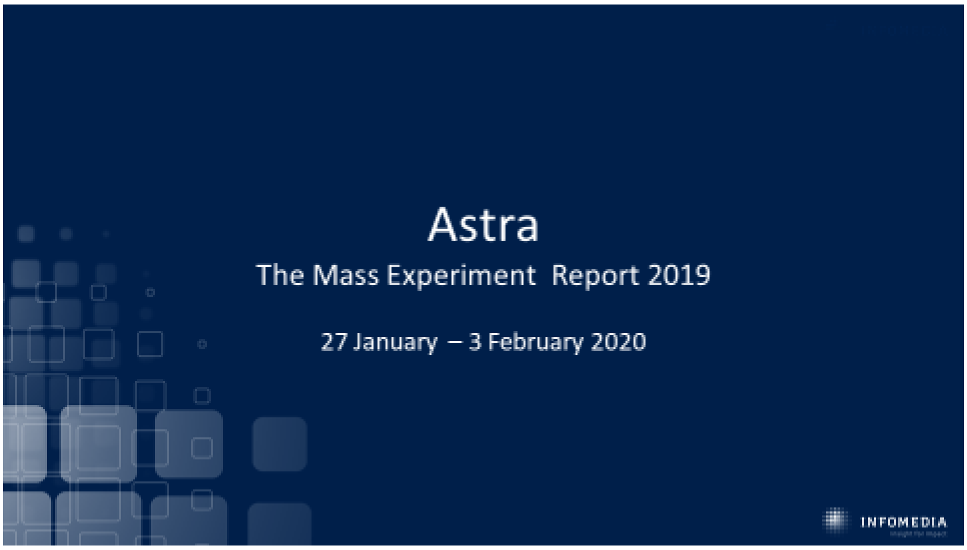

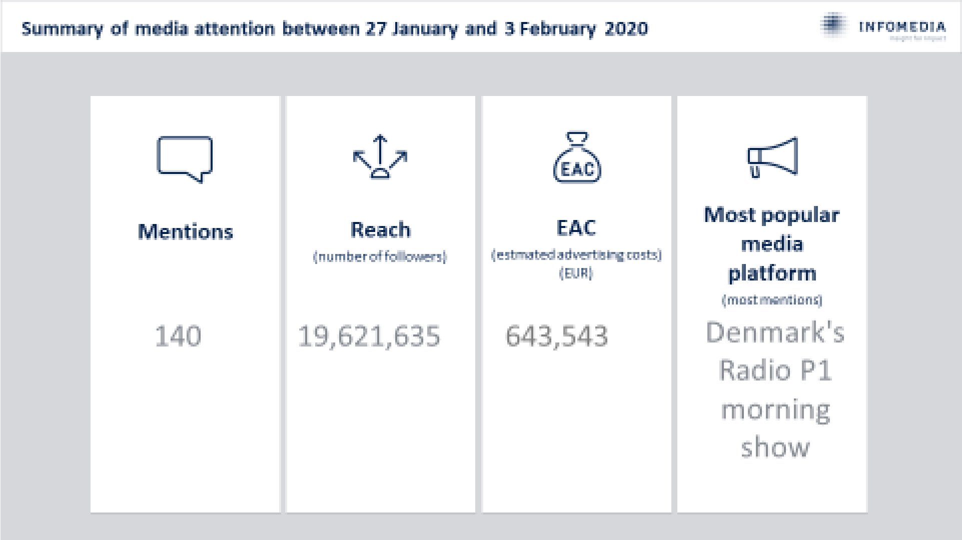

Development in media attention between 27 January and 3 February 2020

INFOMEDIA

Weekly breakdown

Dates		Reach	EAC (EUR)
27 Jan. - 2 Feb.	140	15,821,835	642,542.24
3 Feb-9 Feb	0	0	0

Daily percentage

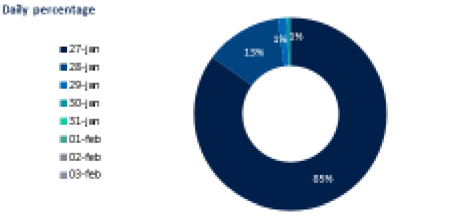

Development as number of mentions and reach per day

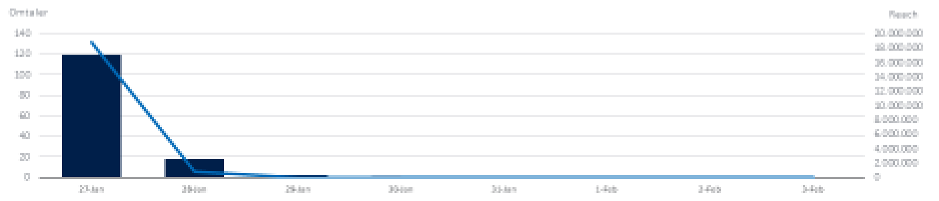

Media attention 27 January to 3 February 2020

INFOMEDIA

Summary of mentions and reach divided by media source

Source	Mentions	Reach	EAC (EUR)
Web sources	59	11,472,235	262,262
Local newspapers	27	1,717,00	37,085
Radio programs	24	1,957,000	27,027
TV programs	11	1,991,000	150,935
News service	6	0	0
Daily newspapers	4	482,800	16,264

Media sources with highest numbers of mentions

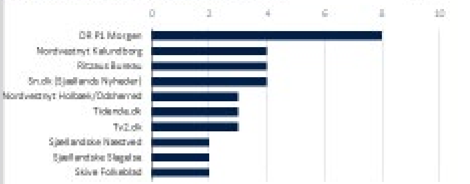

Media sources with greatest reach

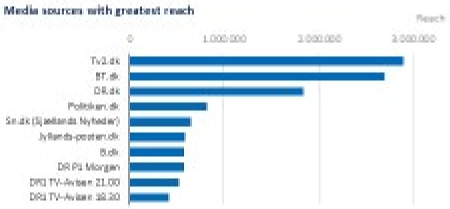

Media sources with highest advertising costs (DKK)

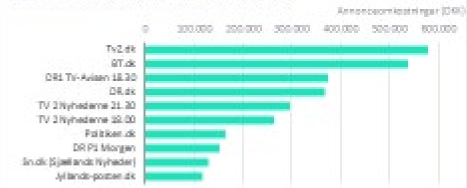

Regional media attention between 27 January and 3 February 2020

INFOMEDIA

Mentions, reach and estimated advertising costs (EUR) per region

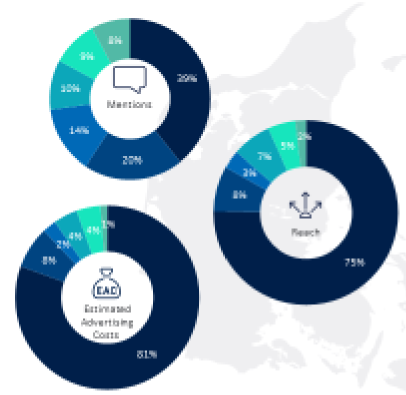

Region of Denmark	Mentions	Reach	EAC (EUR)
Denmark-entire country	55	14,762,228	518,004
Zealand	28	1,558,860	47,752
Mid Jutland	19	628,784	15,538
North Jutland	14	1,346,686	27,424
South Denmark	13	956,722	27,086
Capital City	11	370,355	7,740

Top 10 mentions during the period from 27 January and 3 February 2020

INFOMEDIA

Mentions with highest advertising costs (EUR)

	Headlines (translated from Danish to English)	Media source	Date	Reach	EAC (EUR)
1	The Mass Experiment: nothing pollutes like cigarette butts	DR1 TV-Avisen 18.30	27-01-2020	419,000	50,058
2	Students and teachers collected 112,018 cigarette butts in just a few hours' It is surprising and disgusting'	DR.dk	27-01-2020	1,837,000	49,280
3	The Mass Experiment: Cigarette butts and sweet wrappers top the list of plastic litter	TV 2 Nyhederne 21.30	27-01-2020	332,000	39,649
4	Most plastic dumped in the countryside can't be recycled	BT.dk	27-01-2020	1,345,500	38,095
5	Smokers pollute the countryside with cigarette butts	BT.dk	27-01-2020	1,345,500	38,095
6	Cigarette butts top the list of plastic litter	TV 2 Nyhederne 18.00	27-01-2020	298,000	35,357
7	Plastic litter collected over a distance of 355 kilometer – politician 'deeply concerned' over findings	Tv2.dk	27-01-2020	965,500	25,900
8	Todays overview: Help for Danes in China	Tv2.dk	27-01-2020	965,500	25,900
9	Most plastic dumped in the countryside can't be recycled	Politiken.dk	27-01-2020	820,500	22,010
10	Smokers pollute nature with cigarette butts	Jyllands-posten.dk	27-01-2020	591,500	15,868

Social Executive Summary

27 January to 3 February 2020

INFOMEDIA

Summary of attention from media platforms 27 January to 3 February 2020

INFOMEDIA

Hits per media platform

Media platform	Mentions	Potential reach	Followers
Twitter	27	35.6K	119
Facebook	8	681.7K	1K
Blogs	8	48.2K	0
Fora	2	1.78	0
Instagram	1	-	215

Number of mentions

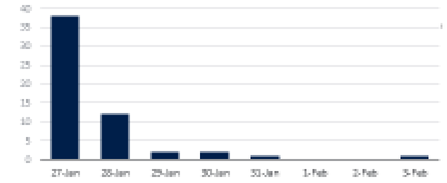

Percentage for each media platform

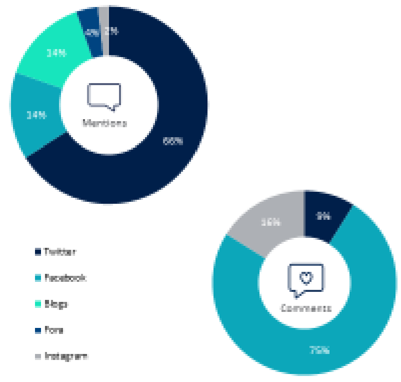

Influencers og media platforms

Top influencers with highest number of comments (likes/comments/shares)

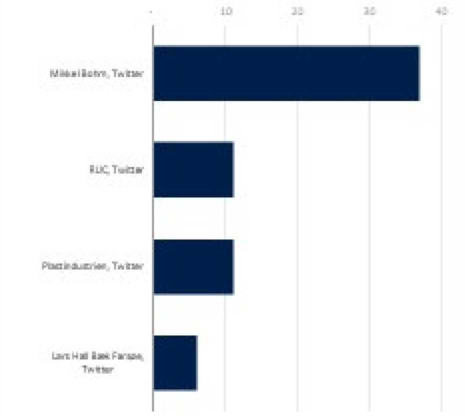

Wordcloud - mentions

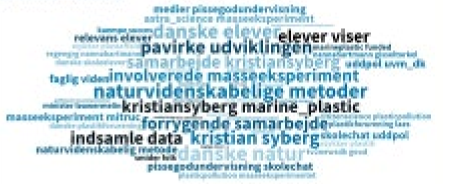

Wordcloud - hashtags

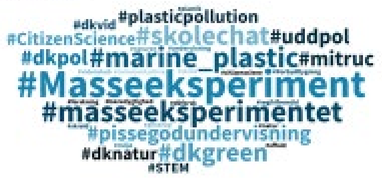

Top 10 mentions *(continued)*

	Extract of mention (translated from Danish)	Influencer	Media platform	Date	Potential reach	Followers
6	Students are motivated and learn more when they can see the application of scientific experiments to everyday life. And that is the case in @astra_science #Masseeksperiment about plastic in nature, that is in focus in all media...	Mikkel Bohm, @MikkelBohm	Twitter	27-01-2020	820	28
7	57.000 Danish students show how they can collect data that can be a game changer to society using scientific techniques......	Astra, @astra_science	Twitter	27-01-2020	406	25
8	...great work by classes 6.a and 6.b who participated in The Mass Experiment in the autumn. The students collected loads of plastic here in Jelsley. Today they are being interviewed by both TV and radio stations.....	Tofbogårdskolen	Facebook	27-01-2020	7.7K	18
9	Not only have Danish students provided researchers worldwide with a unique dataset, they have also participated in a science lesson that has for sure stimulated their enquiring minds and challenged their practical skills #Masseeksperiment	Elsebeth Wahlk, @Elze_beth	Twitter	27-01-2020	407	11
10	We are grateful to be a part of The Mass Experiment. Plastic packaging should be designed so that it can be sustainable following EU guidelines, but as a society we also must take responsibility to change our habits when we are out and about.	Plastindustrien, @plastindustrien	Twitter	27-01-2020	1.5K	11

Top 10 mentions

Most popular mentions

	Extract of mention (translated from Danish)	Influencer	Media platform	Date	Potential reach	Followers
1	Students collected 374,082 pieces of plastic litter - 112, 018 of them were cigarette butts. The Danish countryside is full of plastic litter – cigarette butts top the list. See the five most frequent finds......	TV 2 Nyhederne	Facebook	27-01-2020	614.7K	639
2	In just 2 weeks, Danish students collected 374,082 bits of plastic in Danish nature! Cigarette butts were the greatest find at a shocking 112,018	Videnskab.dk	Instagram	27-01-2020	-	215
3	Thousands of students participated last year in a huge plastic litter collection. Their finds are analysed and almost one third is a cigarette butt.	Miljøstyrelsen	Facebook	27-01-2020	17.5K	170
4	Cool students from Linde High School collect 5300 pieces of trash. The Mass Experiment is a huge national experiment in which 57, 000 students have collected 370,000 pieces of litter in Danish nature. The school that found ...	Herlev Kommune	Facebook	28-01-2020	1.6K	122
5	Empty crisp bags, sweet wrappers and especially cigarette butts are found everywhere as litter in Denmark. Almost one third of litter is a cigarette butt.	RUC	Facebook	27-01-2020	10.8K	67
